# Sequential Bottlenecks Drive Viral Evolution in Early Acute Hepatitis C Virus Infection

**DOI:** 10.1371/journal.ppat.1002243

**Published:** 2011-09-01

**Authors:** Rowena A. Bull, Fabio Luciani, Kerensa McElroy, Silvana Gaudieri, Son T. Pham, Abha Chopra, Barbara Cameron, Lisa Maher, Gregory J. Dore, Peter A. White, Andrew R. Lloyd

**Affiliations:** 1 Inflammation and Infection Research Centre, School of Medical Sciences, University of New South Wales, Australia; 2 School of Biotechnology and Biomolecular Sciences, University of New South Wales, Sydney, Australia; 3 Institute of Immunology and Infectious Diseases, Murdoch University, Perth, Western Australia, Australia; 4 School of Anatomy and Human Biology, Centre for Forensic Science, University of Western Australia, Perth, Western Australia, Australia; 5 The Kirby Institute, University of New South Wales, Sydney, Australia; University of Southern California, United States of America

## Abstract

Hepatitis C is a pandemic human RNA virus, which commonly causes chronic infection and liver disease. The characterization of viral populations that successfully initiate infection, and also those that drive progression to chronicity is instrumental for understanding pathogenesis and vaccine design. A comprehensive and longitudinal analysis of the viral population was conducted in four subjects followed from very early acute infection to resolution of disease outcome. By means of next generation sequencing (NGS) and standard cloning/Sanger sequencing, genetic diversity and viral variants were quantified over the course of the infection at frequencies as low as 0.1%. Phylogenetic analysis of reassembled viral variants revealed acute infection was dominated by two sequential bottleneck events, irrespective of subsequent chronicity or clearance. The first bottleneck was associated with transmission, with one to two viral variants successfully establishing infection. The second occurred approximately 100 days post-infection, and was characterized by a decline in viral diversity. In the two subjects who developed chronic infection, this second bottleneck was followed by the emergence of a new viral population, which evolved from the founder variants via a selective sweep with fixation in a small number of mutated sites. The diversity at sites with non-synonymous mutation was higher in predicted cytotoxic T cell epitopes, suggesting immune-driven evolution. These results provide the first detailed analysis of early within-host evolution of HCV, indicating strong selective forces limit viral evolution in the acute phase of infection.

## Author Summary

Primary hepatitis C (HCV) infection is typically asymptomatic and commonly results in persistent infection. The characteristics of early infection remain undefined. Four subjects were studied longitudinally from within a few weeks of transmission until resolution of outcome, via a full genome analysis of viral evolution. In the *acute* phase (<100 days post-infection) there were two periods with a major reduction in genetic diversity (i.e. a bottleneck) irrespective of subsequent clearance (n = 2) or chronic infection (n = 2). The first bottleneck was associated with transmission, with generally only one ‘founder’ virus successfully establishing infection. The second occurred following the primary peak in viraemia, concomitant with seroconversion, approximately 100 days post-infection. In the subjects who became chronically infected, the second bottleneck was followed by emergence of a new cluster of variants, which evolved from the founder(s), and carried only a small number of mutated residues that reached fixation. Some fixations occurred in known targets of CD8 cytotoxic T cell and neutralizing antibody responses. These results indicate a common evolutionary pattern, independent of disease outcome in the *acute* phase of HCV infection, with strong signatures of selective pressures driving the transition into chronic infection. These novel data will inform preventative vaccine strategies.

## Introduction

Hepatitis C virus (HCV) infection is a major cause of chronic liver disease, resulting in substantial morbidity and mortality worldwide, with between 123 and 170 million persons infected [1]. Transmission is predominantly via blood-to-blood contact associated with contaminated injection devices. Infection persists in approximately 70% of acute cases, leading to chronic hepatitis, and ultimately cirrhosis and the associated complications of liver failure and hepatocellular carcinoma [2]. The outcome of primary HCV infection is driven by the interplay between rapid viral evolution and host adaptive immune responses [3], [4]. Analogous to other RNA virus infections, development of effective vaccines and antiviral treatments has been constrained by the ability of these viruses to swiftly overcome evolutionary pressures such as host immunity and antiviral drugs [5]–[7].

The rapid rate of evolution in RNA viruses is driven by a highly error-prone RNA-dependent RNA polymerase (RdRp). For HCV, the estimated mutation rate is 1.2×10^−4^ substitutions per site per infected cell [8], and with a half-life of 3–5 hrs, it is estimated that at least 10^12^ particles are generated per day [9]. With these rapid kinetics, at least 10^9^ variants with single- and double-nucleotide changes are likely to arise in each individual multiple times daily [10]. However, the observed viral complexity is much less than this prediction, largely due to reduced fitness of mutated variants [11]. While the error-prone viral replicase results in a continuous supply of new viral variants (genetic drift), purifying selection generated from host immune pressure and viral fitness results in culling and preferential selection of certain variants. When selection overbalances drift, a genetic bottleneck occurs (i.e. evolutionary events resulting in a reduction in genetic variation due to extinction of a significant proportion of the viral variants).

Very little quantification of the within-host evolutionary dynamics of human RNA viruses has been reported, and limited information exists from experimental evolution systems [12], and from viruses infecting animals. For RNA viruses, the effective population size - broadly equated with the number of variants that will contribute genes to the next generation, a key parameter in viral evolution - is smaller than the census size. This discrepancy is often seen at the epidemiological level as result of a strong transmission bottleneck. Well-characterized within-host genetic bottlenecks have been observed upon transmission of a number of RNA viruses, including HIV [13]–[15]. A low effective population size has important consequences for viral evolution, as at small effective population numbers (*N_e_*), random processes (i.e. genetic drift) predominate over deterministic ones (i.e. selection) [16]. Genetic bottlenecks severely limit viral diversity and potentially limit replicative fitness of the resultant virus (reviewed in [12]). After a bottleneck event, RNA viruses undergo rapid evolution, leading to accumulation of deleterious mutations and the occurrence of rare fit variants, which can rapidly reach fixation and dominate the next population.

Following a genetic bottleneck, within-host evolution of rapidly mutating viruses can be characterized by selective sweeps (i.e. the reduction or elimination of variants in the viral population as the result of strong selection pressures), with only a few variants emerging to dominate the future population. This phenomenon is common at the host population level, such as in influenza [17], and has been documented within-host for HCV [18], [19] and HIV [20]. The extent to which positive selection and random genetic drift contribute to the within-host evolution of HCV, and whether selective sweeps observed late in primary infection are related to genetic bottleneck events, remain to be resolved.

A major challenge in studies of the within-host evolution of RNA viruses lies in the capacity to detect low frequency viral variants. Standard cloning techniques, and more recently single genome amplification, followed by sequencing have been used to detect variants present at frequencies as low as 10–15% [15], [21], [22]. Next generation sequencing (NGS), despite high technical errors [23], allows detection of rare variants present at less than 1% of the population [24]–[33]. Data analysis tools have now improved the ability to differentiate true biological variation from technical error [34], [35], and also enabled reconstruction of genomic regions of individual variants from short NGS reads [34], [36].

The early evolution of HCV has been investigated to date only in limited case series [18], [30], [35], [37]–[40], and with a strong bias towards symptomatic cases, who represent a small minority of those with acute infection, and who are known to have an increased likelihood of clearance [3], [4]. Few studies have investigated early infection in a longitudinal fashion [30], [35], [39], [41]. In these cases the frequency, distribution, and timing of viral mutations has been characterized only in limited regions of the virus and with low sensitivity in the detection of rare variants.

Here we report a comprehensive analysis of longitudinal collected samples from four subjects identified very early in asymptomatic acute HCV infection. The aim was to quantify the number of successfully transmitted founder viruses, and to characterize the diversity and complexity of viral population across the genome over the course of the primary infection leading to clearance or chronicity.

## Results

### Subjects

**Figure 1:**
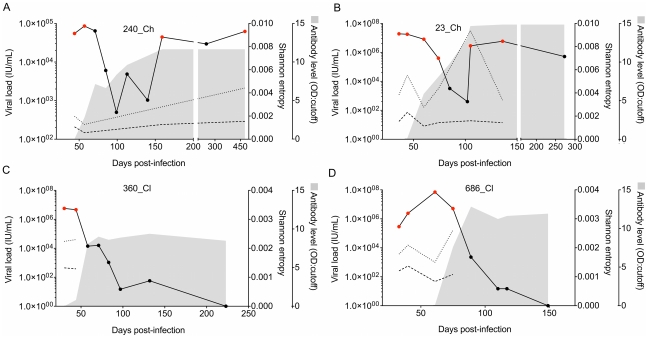
RNA level, Shannon entropy, and antibody titers over time for the four early infection subjects. Panels A and B show two subjects who developed chronic infection (240_Ch, 23_Ch) followed from pre-seroconversion timepoints. Panels C and D show the infection dynamics for two subjects who ultimately cleared the infection (360_Cl, 686_Cl). Red dots represent viremic time points analyzed via next generation sequencing (NGS). The solid line represents the RNA level. The dashed and dotted lines represent the interpolation of the Shannon entropy calculated across the genome at each time point using NGS data. Entropy was calculated using all mutated sites (dotted line), or with only non-synonymous sites (dashed line). The shaded area represents semi-quantitative estimates of the anti-HCV antibody titer (OD: cut-off). Note the varied ranges in the x- and y-axes.

Four newly viremic seronegative subjects were studied (Figure 1, Table 1, Table S1). Subjects were enrolled in the Hepatitis C Incidence and Transmission Study (HITS) cohort, and had tested negative for HCV antibodies and RNA within 3–6 months prior [42], [43]. The estimated days post-infection (DPI) at enrolment ranged from 30–45 days (Table 1). Two subjects cleared the infection (686_Cl, 360_Cl), and the other two progressed to chronic infection (23_Ch, 240_Ch). A single viral genotype (GT) was detected in each subject: two with GT1a (686_Cl, and 23_Ch), and two with GT3a (360_Cl, and 240_Ch). Figure 1 shows the HCV RNA levels and HCV-specific IgG antibody titers (estimated as the optical density to cut-off ratio in the enzyme immunoassay) over the course of infection.

**Table 1:** Subject characteristics, number of founder viruses, and estimates of the time since the most recent common ancestor (tMRCA).

Subject ID	Sex	Disease outcome	GT	Age	First sampling timepoint (DPI^a^)	Observed duration of viraemia (DPI)	Timepoints analysed via NGS^b^	Length of genome investigated	Maximum entropy (full genome^c^)	Number of founder viruses	Poisson estimated tMRCA (95% CI) d
686_Cl	F	Cleared	1a	25	33	117	4	9172	0.010414	1	35 (26,47)
23_Ch	M	Chronic	1a	25	36	304	6	9138	0.002323	2	44 (24, 64)
360_Cl	M	Cleared	3a	29	30	223	2	5992	0.003190	1	34 (20, 57)
240_Ch	M	Chronic	3a	24	44	477	4	9226	0.001546	1	34 (19,48)

a Estimated days post-infection (DPI).

b Next Generation Sequencing.

c Calculated across the genome for non-synonymous substitutions.

d Mean value from the estimates of the time since most recent common ancestor (tMRCA) estimated from reconstructed viral variants across the genome in windows of 400 nt calculated according to a Poisson model of viral mutation (45). Confidence intervals are the 5^th^ and 95^th^ percentiles of the total range of confidence intervals estimated in each of the windows analyzed across the genome.

For the purposes of this analysis, the time course of the primary infection was divided into three phases: i) *transmission*; ii) an *acute* phase (designated as <100 DPI); and iii) a *pre-chronic* phase (designated as >100 DPI) leading to clearance or chronicity at six months post-infection).

### Evolutionary dynamics of viral diversity over the course of the infection

**Figure 2:**
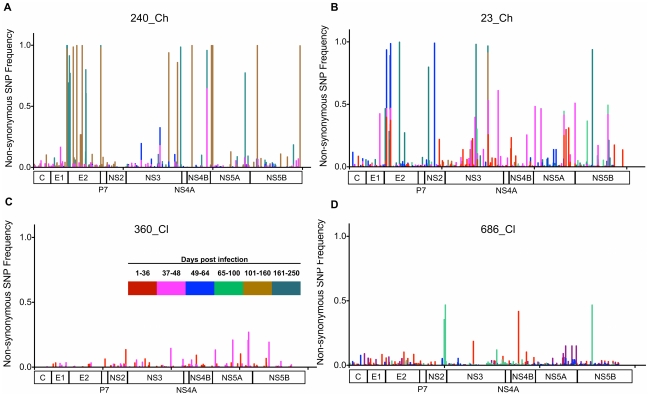
Distribution of the non-synonymous substitutions detected across the genome over the course of the infection. Panels A and B show the distributions of non-synonymous substitutions in two subjects who developed chronic infection (240_Ch, 23_Ch). Panels C and D show two subjects who ultimately cleared the infection (360_Cl, 686_Cl). Each panel shows the longitudinal analysis of the distribution of non-synonymous substitutions for each subject. In subjects who cleared the infection, substitutions sporadically emerged at low frequency (<50%). In the two subjects that developed chronic infection, several substitutions across the full genome reached fixation (>99%). Colors represent the time course post-infection (see legend).

NGS, standard cloning and bulk (consensus) sequencing were performed on viremic samples collected longitudinally from each of the subjects (Figure 1). On average, 81,900 reads with average read lengths of 358 bp were generated per subject per timepoint, giving an average coverage depth of 3,093 (Table S2). Single nucleotide polymorphisms (SNP) analysis revealed between 160 and 460 nucleotide substitutions per timepoint. Over the course of the infection more than half of the substitution events occurred at frequencies of <1%, except in subject 240_Ch (38%). Non-synonymous substitutions constituted 40%–57% of the total and were distributed across the genome (Figure 2). Within the first 60 DPI, the prevalence of non-synonymous substitutions was approximately half of the total (50–57%), except in subject 686_Cl (34%). Subject 686_Cl showed the highest prevalence of substitutions with a frequency <1% (76% during the first 60 DPI). In the two subjects who became chronically infected (23_Ch and 240_Ch), the prevalence of substitutions was significantly higher than in subjects who ultimately cleared the infection (686_Cl and 360_Cl). In subjects that progressed to chronicity, some substitutions reached frequencies above 99% (i.e., fixations; see also below).

Shannon entropy (SE) was calculated from the frequencies of nucleotide substitutions measured across the genome. During the *acute* phase, the SE measures indicated a general decline in viral diversity. This decline did not follow the HCV RNA kinetics. Similar patterns were observed for SE calculated with all substitutions, or with only non-synonymous changes. In two subjects (686_Cl, 23_Ch) at least two pre-seroconversion timepoints were sampled within a window of eight weeks, allowing an accurate assessment of very early HCV evolution, and revealing a peak in viral diversity in temporal proximity to the viremic peak. In the two subjects who ultimately became chronically infected (23_Ch and 240_Ch), SE increased in the *pre-chronic* phase, which also featured a continued increase in HCV RNA level (Figure 1). The observed patterns of change in SE measured across the full genome were not consistently observed in the 10 protein-encoding regions, clearly indicating a non-uniform evolution of HCV across the genome and between subjects.

### Transmission - First bottleneck

To test the hypothesis that a single virus establishes infection upon transmission, a statistical model, PoissonFitter [44] was used to examine whether the viral population had a star-like phylogeny with a Poisson distribution [22]. For this analysis reconstructed viral variants were obtained from NGS reads using a Bayesian statistical tool, ShoRAH [34], [36]. Firstly, viral haplotypes were reconstructed from NGS data at the first viremic timepoint segregated into 400 nt windows across the genome (see Table S3, including the number of variants reconstructed per window). Secondly, reconstructed haplotypes of the E1/HVR1 (871 nt) and partial E2 (932 nt) regions were utilized, along with the corresponding E1/E2 clonal sequences (Table S3). In the second dataset, only variants with a frequency greater than 2.5% were included in this analysis. This cut-off was derived through a validation analysis for the method of reconstruction of viral haplotypes (of length > 400 nt) using a mixed sample of four plasmid E1/E2 clones derived from one subject (see Supporting Text S1 for details). By definition, the sequence of the founder was identified as being: i) the most prevalent variant; and ii) identical to the consensus sequence of the viral population at the first time point [15], [22].

**Figure 3:**
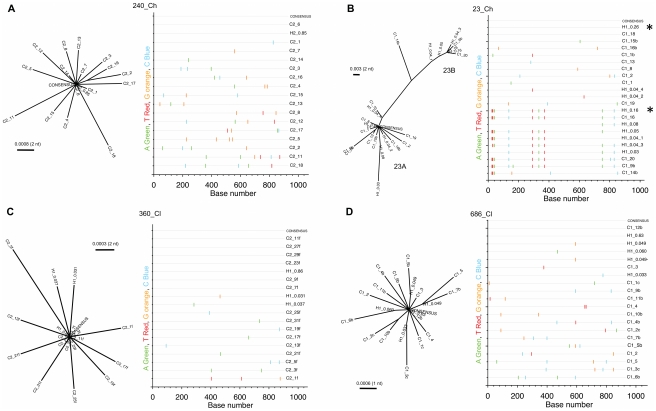
Founder virus analysis based on partial E2 region of the viral genome. Panels A and B show the analyses for two subjects who developed chronic infection (240_Ch, 23_Ch) followed from pre-seroconversion timepoints. Panels C and D show the analysis for two subjects who cleared the infection (360_Cl, 686_Cl). Phylogenetic reconstructions and highlighter plots are shown, illustrating the genetic relatedness between HCV variant sequences. Names of each sequence are labeled with a letter (H for haplotype, and C for clone), with the first number representing the sampling timepoint and with the second number representing either the prevalence of the haplotype or the clone number. The phylogenetic trees of subjects 686_Cl, 360_Cl and 240_Ch (panels A, C, D) are consistent with an infection arising from a single founder. The fit with a Poisson model is also consistent with a single founder (p-value > 0.1, see text). As shown by the highlighter plots, founder viruses are identified as the consensus sequence and coincided with the most prevalent variant reconstructed from NGS data, (e.g. for subject 686_Cl H1_0.60 is identical to the consensus sequence and to clone C_12b). The highlighter plots also show the random distribution of mutated sites with respect to the founder sequence (master), which is consistent with a star-like phylogeny. The phylogenetic analysis in 23_Ch (panel B) is consistent with an infection originated from two founder viruses (indicated with an asterisk in the highlighter plot) giving rise to two major clusters, 23A and 23B. This is consistent with the rejection of the Poisson model (p-value = 0). Phylogenetic trees were obtained using PhyML, with Maximum Likelihood methods using a GTR model of substitution as suggested by model testing.

Both the PoissonFitter tests and phylogenetic analyses indicated that the HCV infection in three subjects (686_Cl, 360_Cl, and 240_Ch) was successfully established by a single variant (Figures 3, S1, S2 and Table S3). For these subjects, the highlighter plots show the random distribution of SNPs across the sequences, again consistent with a star-like distribution of variants arising from a single founder. By contrast, Poisson analysis of the remaining subject (23_Ch) indicated that more than one virus established the infection. Phylogenetic analyses of E1, E2 and NS3 regions of subject 23_Ch indicated that at least two viruses had established the infection, designated 23A_F_ and 23B_F_ (Figures 3 and S1, Table S3). These founders generated two major clusters of variants, which were named after the founder variants. These two founder variants were identified with comparable prevalence in both E1/HVR1 (Figure S1) and E2 regions (Figures 3). The average genetic difference between these two dominant variants over E1/E2 regions was 1.3%. In support of these results, the founder variants were also identified via clonal sequencing in both E1/HVR1 (Figure S1) and E2 regions (Figure 3). For 23_Ch, the calculation of the exact number of founder viruses was confounded by the presence of several variants that appeared to have arisen from recombination events between two viruses from each cluster (see for example, variant C1_14b in Figure 3).

A total of 2-5 variants (including the founders) were detected with a frequency above 2.5% in 240_Ch, 686_Cl, and 360_Cl at the first viremic timepoint. Subject 23_Ch presented the most diverse repertoire, with nine variants present in the E1/E2 region. These results, in combination with the fact that >50% of substitutions were present at a frequency <1% (see above), indicate that early HCV evolution was characterized by a large distribution of low frequency variants.

The time since the most recent common ancestor (tMRCA), which is estimated with PoissonFitter, provides an empirical measure of the likely timing of the transmission event [22], [45]. The estimated tMRCA from independent analyses of E1/HVR1 and partial E2 regions closely matched the DPI estimated from the seroconversion timepoint (Tables 1 and S3). The tMRCA estimated from the founder analyses in windows of 400 nt across the full genome also matched the DPI estimated from the seroconversion timepoint (Table S3). As expected, estimates of the tMRCA in 23_Ch from genomic regions with evidence of at least two founders were longer than those estimated from the seroconversion timepoint, indicating that the observed diversity was not likely to be generated by a single founder.

### Acute phase - Second bottleneck

Analysis of the viral dynamics in the *acute* phase of infection (i.e. <100 DPI) for each subject revealed a second genetic bottleneck event brought about by the extinction of several variants. Evidence for this second bottleneck was supported by: i) the decline in SE for all four subjects at the end of the *acute* phase; ii) a decline in viral diversity evidenced by phylogenetic analysis; and iii) a decrease in the effective population size estimated from viral sequences in the two subjects who developed chronic infection.

**Figure 4:**
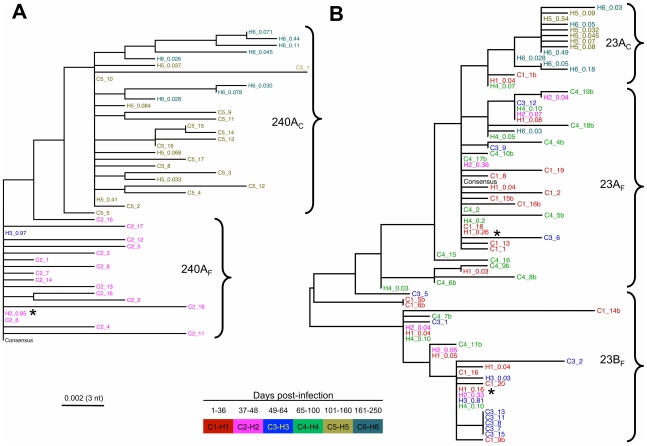
Evolutionary dynamics of HCV variants over the partial E2 region of the genome. Sequence analyses of the two subjects who developed chronic infection, 240_Ch (A), and 23_Ch (B) revealed the presence of selective sweeps. These sweeps led to the emergence of new variants that replaced the founder viruses (identified with an asterisk). Phylogenetic trees (left panels in A and B) display nucleotide sequences of reconstructed haplotypes derived from NGS data and clonal sequences. Names of each sequence are labeled with a letter (H for haplotype and C for clone), with the first number representing the sampling timepoint and with the second number representing either the prevalence of the haplotype or the clone number. Colors are also used to portray the sampling timepoint (see legend). Infection dynamics for subject 240_Ch are consistent with a single founder, identified with the most prevalent strain of cluster 240A (H2_0.85 and H3_0.97 at time-points 2 and 3 respectively), with clone C2_6, and with the consensus of the sequences from time-point 1. The *pre-chronic* phase (corresponding with the color-coded time ranges 5 and 6) of infection shows the emergence and dominance of a new subgroup of viruses, designated 240B. 23_Ch has at least two founder viruses that successfully initiated the infection (H1_0.26 and H1_0.16 within the two clusters 23A_F_ and 23B_F_, respectively), A new cluster 23A_C_, termed after the dominant variant H5_0.54, emerged in the *pre-chronic* phase and replaced cluster 23A_F_. Trees are calculated using Maximum Likelihood method (implemented in PhyML).

Phylogenetic reconstruction of partial E2 nucleotide sequences showed a decline in viral diversity at the end of the *acute phase* (Figure 4). In 240_Ch, infection was successfully initiated with a single founder, 240A_F_, identified here as the most prevalent variant (85% of the population) of the cluster (termed 240A_F_ after the founder) observed in the *acute* phase (Figures 3 and 4). The early increase in prevalence of the founder variant (up to 97% by 57 DPI) and the absence of minor viral variants, are consistent with the observed decline in SE and increase in HCV RNA level. The two founders identified in 23_Ch differed only at three residues in the E2 region (402, 443 and 446). Initially, these founders were present at comparable levels within the viral population (26% and 16% respectively, Figure 4). However, by 60 DPI, the founder variant within cluster 23A_F_ was undetectable - concomitant with seroconversion and with the decline in HCV RNA level between 44 DPI and 60 DPI (Figure 4). The prevalence of the second founder variant, 23B_F_ remained reasonably stable within the cluster until 60 DPI, whereas by 74 DPI its frequency was greatly diminished, consistent with the overall reduction in HCV RNA level. Similar patterns of decline in viral diversity were also observed by phylogenetic analyses in other regions of the same subjects (E1/HVR1, Figure S3 and NS3, Figure S4) and also in the other two subjects (not shown).

**Figure 5:**
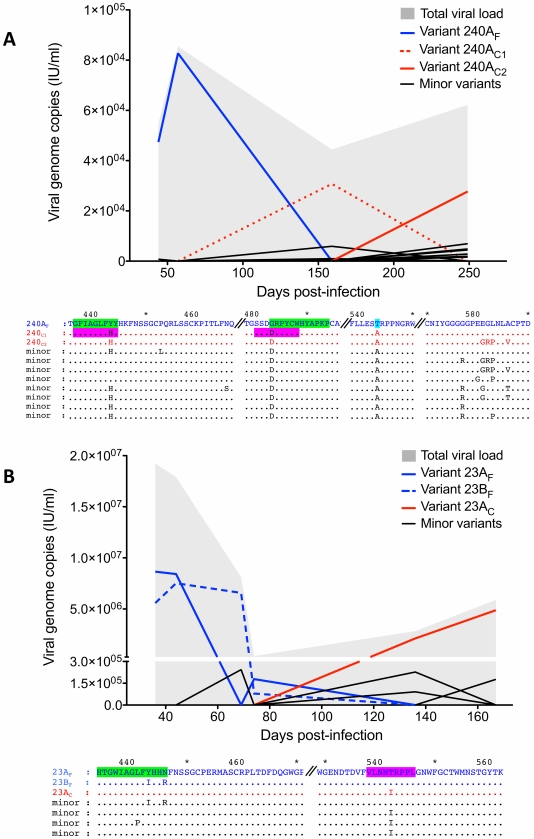
Evolution of the partial E2 region of individual HCV variants at the amino acid level. The plots in A (subject 240_Ch) and B (23_Ch) show the dynamics of the individual viral variants over time. In 240_Ch, infection was initiated with one founder variant, 240A_F_, which was then replaced sequentially by two related variants, 240A_C1_ and 240A_C2_, respectively. In 23_Ch, at least two founders initiated infection, 23A_F_ and 23B_F_, which both dominated the early phase of infection before being replaced by a new variant in the *pre-chronic phase* of infection (23A_C_). The y-axis shows the contribution of each variant with respect to the RNA level. Below each graph is an amino acid alignment indicating the distinguishing residues for the different variants. The location of putative CTL (pink shading) and B cell (green shading) epitopes, and mutations with previously recorded viral fitness costs (light blue shading) are indicated. All the identified epitopes within this region carried at least one amino acid change. Two of these mutations (G483D for 240_Ch and T542I for 23_Ch) generated CTL epitopes with reduced binding CTL affinity, and both subjects showed a substitution at position Y443, known to be within a B cell epitope [47] - all of which is suggestive of immune escape. In addition, in 240_Ch a potential fitness cost associated mutation was observed at T543A [48].

The evolutionary trajectories of individual viral variants at the amino acid level in the partial E2 region (Figure 5) and E1/HVR1 (data not shown), also showed the dominance of two distinct viruses for 23_Ch (23A_F_ and 23B_F_) and one for 240_Ch (240A_F_) in the *acute* phase. Minor variants emerged during this phase with the majority receding below the limit of detection.

**Figure 6:**
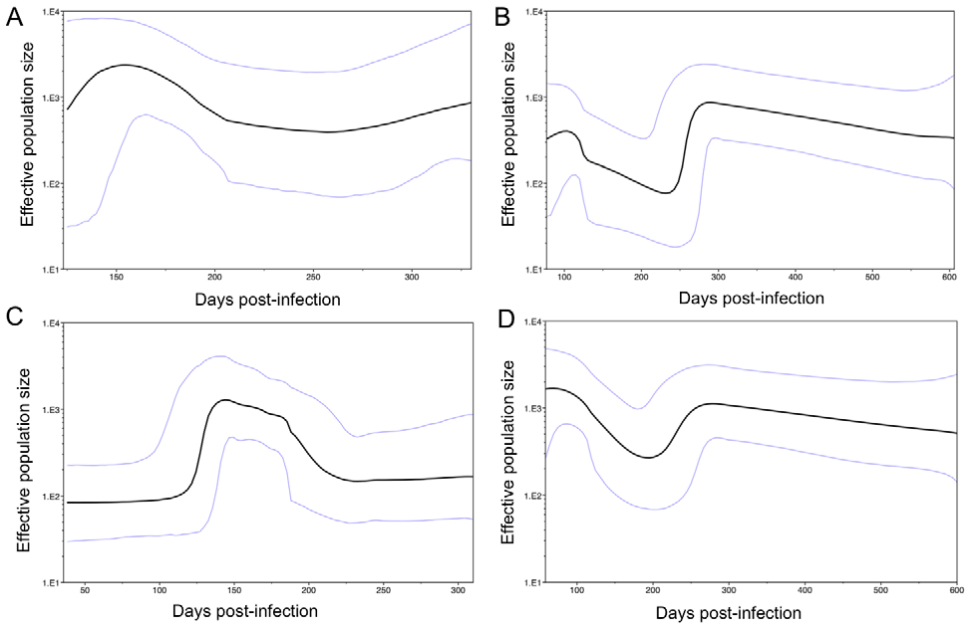
Demographic reconstruction of the viral populations. Demographic reconstruction from E1/HVR1 (A,B) and E2 (C,D) sequences for subjects who developed chronic infection, 23_Ch (A,C) and 240_Ch (B,D). In both subjects, and in both genomic regions, the founder effect and sequential bottleneck events are evident. The estimated effective population size (Nτ, the product of the effective population size and generation length in days) had a peak value of the order of 10^3^, and then decreased to values of the order of 10^2^. The longer estimate of tMRCA for 23_Ch when compared to those in Table 1 is likely to be due to the presence of two founder viruses in this subject.

A decrease in the effective population size also provided evidence of sequential bottleneck events. A coalescent demographic reconstruction of the viral population within each host, using the Bayesian skyline plot [46] on E1/HVR1 and partial E2 regions (Figure 6) revealed a decline in diversity (measured as Nτ, the product of the effective population size and generation length in days) during the *acute* phase of infection in both subjects who became chronically infected (23_Ch and 240_Ch). The estimates of the time of infection from this analysis were consistent with those found via the Poisson model (Table S3). Demographic reconstruction for subjects who cleared infection (360_Cl and 686_Cl) showed little variation in effective population size over the course of the infection (Figure S5), which is likely to reflect the short time interval covered by the available samples.

### Pre-chronic phase - Selective sweep

The *pre-chronic* phase (i.e, >100 DPI) featured selective sweeps, with the new variants arising from the preceding viral population in the *acute* phase, showing remarkably few substitutions reaching fixation in the new population. In subject 240_Ch, following the decline in HCV RNA level at 140 DPI, a new cluster of variants, termed here 240A_C_, figure 4) replaced the 240A_F_ cluster via a selective sweep brought about by the second bottleneck event. Similarly, in 23_Ch, the recrudescence of viremia in the *pre-chronic* phase was associated with the emergence of a new cluster, 23A_C_, which evolved only from cluster 23A_F_ via a selective sweep (Figure 4b). In both subjects a single variant dominated the *pre-chronic* phase population, accounting for 74% of the total HCV RNA level in subject 23_Ch, and approximately 50% in 240_Ch. Further evidence in support of the establishment of a new viral population in the *pre-chronic* phase was provided by the increase in effective population size following the second bottleneck, which was particularly evident for 240_Ch (Figure 6).

Fixation (>99%) was observed in a limited number of sites in association with the selective sweep (Figure 2). Fixation occurred at two sites in 23_Ch by 136 DPI (E2, 542; NS3, 1498) and at 12 sites in 240_Ch by 159 DPI (E1, 372; E2: 443, 483 and 543; NS2, 750; NS3: 1509, 1606; NS4b: 1768; NS5a: 1985, 1999; NS5B: 2497 and 2973). Further evolution was observed with additional fixation sites occurring by 304 DPI in NS2 (856) and NS5B (2629), indicating the presence of a further selective sweep event. In subject 240_Ch a further selective sweep event occurred by 477 DPI, with fixations observed in E2 (400, 405, 408, 583), NS3 (1641), and NS5a (2361).

### Evidence of immune driven selection

In order to determine whether non-synonymous substitutions were likely to be driven by cellular and humoral immune responses, we identified the location of predicted, as well as experimentally-verified, epitopes across the full genome for each subject (Table S4). Analysis of HLA class I restricted cytotoxic T cell (CTL) epitopes revealed a total of 62 amino acid substitutions (29 in 23_Ch, 12 in 240_Ch, 8 in 686_Cl, and 13 in 360_Cl) which were found to be located in HLA-restricted epitopes (Table S4, Figure 5). Twenty-four of these substitutions resulted in mutant epitopes with a lowered predicted binding score, suggesting that these substitutions potentially conferred a CTL immune escape phenotype (Table S5). In 23_Ch, all four sites that reached fixation in the *pre-chronic* phase lay within HLA-restricted epitopes. In 240_Ch, of the 18 sites that reached fixation in the *pre-chronic* phase, nine lay within CTL epitopes.

Figure 5 shows the location of the E2 mutations within putative CTL and B cell epitopes in the different variants in subjects 23_Ch and 240_Ch, as well as mutations previously shown to be associated with viral fitness costs. All the identified epitopes within this region carried at least one amino acid change. Two of these mutations (G483D for 240_Ch and T542I for 23_Ch) generated CTL epitopes with reduced binding affinity, thus indicating potential immune escape (Table S4). Interestingly, both subjects also showed a substitution at position 443 (Y443I for 23_Ch and Y443H in 240_Ch) known to be within a B cell epitope [47]. In 240_Ch a mutation at 543 was observed (T543A). Mutations at this site have been reported to alter the yield of precipitated E1/E2 proteins [48].

Comparison between SE (based on all substitutions) at sites of predicted CTL epitopes, with sites at which no epitopes were predicted, revealed significant differences for each subject (Mann Whitney; all p<0.01). In three subjects (excluding 686_Cl who had rare HLA-B alleles, and hence limited information available for epitope prediction), the distribution of SE values based only on non-synonymous substitutions, revealed that epitope regions carried higher SE values (Mann Whitney; p<0.01). These results support the hypothesis that viral evolution is influenced by the host cellular immune response, as has been shown in chronic infection [49]–[52].

## Discussion

This comprehensive examination of the pattern and kinetics of viral evolution across the HCV genome has revealed that early primary HCV infection features at least two significant genetic bottleneck events. The first occurred during or immediately after transmission, where only one or two founder viruses successfully initiated infection. The second occurred within 100 DPI, preceding a decline in HCV RNA level, and in temporal proximity to seroconversion. This second bottleneck was followed either by clearance, or by a selective sweep with new variants emerging from the founder virus to dominate the *pre-chronic* phase of infection. These quantitative analyses of early events in primary HCV infection suggest that strong selective pressures limit viral evolution both during transmission and early infection.

The demonstration here of a single founder virus in three of four IDU-transmitted HCV cases is comparable to studies in HIV, which have shown that the majority of subjects with mucosal transmission had a single founder virus [14], [15], [22], [53], [54]. Interestingly, HIV transmission via IDU was more commonly associated with multiple viruses, although still less than five founders [14]. Investigation of HIV Envelope sequences in 20 heterosexual transmission pairs revealed that the founder variant in the recipient comprised only a small fraction of the viral variants in the donor (i.e. <5%) [55]. The only previous study in HCV using NGS for founder analysis revealed that a small inoculum could explain the limited diversity observed in early infection in two genomic structural regions [30]. For both HIV and HCV, it remains unclear whether the transmission bottleneck is attributable to a low number of variants being transferred between hosts, or is the result of early evolutionary events where a larger number of strains are rapidly eliminated due to varying fitness constraints. As the two founders identified in subject 23_Ch showed relatively limited diversification from each other, it is also plausible that a single founder underwent very rapid evolution within the first few days post-infection in this case.

Very little is known about the phenotypic characteristics of founder viruses in HCV. The Envelope sequences of founder viruses in HIV consistently predict CCR5 tropism, concordant with the evidence for utilization of this co-receptor for infection of tissue macrophages and dendritic cells in the mucosa [56]. A recent phenotypic analysis of HCV variants emerging in HCV-infected liver transplant recipients indicated that strains with increased viral entry efficiency and lower neutralizing affinity were preferentially selected in the post-transplant phase [57]. In the present study, one of the subjects (23_Ch) was infected with at least two founder viruses that differed at only three sites within the envelope region. Interestingly, residues 443 and 446 lie within, or in close proximity to, one of the major CD81 binding domains, spanning residues 436 – 443 [58], suggesting that founder variant 23B_F_ may have had better infectivity allowing it to out-compete variant 23A_F_ in the *acute* phase. Phenotypic analysis of these two variants is currently underway. On close analysis of some of the minor variants within subject 23_Ch, evidence of potential recombination was identified between the two founder variants. Recombination has been described in RNA viruses, but with limited evidence in HCV (reviewed in [59]). However, as these putative recombinant strains are closely related, it is difficult to distinguish a recombination event from convergent evolution, where closely related variants incorporate common mutations due to shared selective pressure [60]. Furthermore, PCR amplification could also cause recombinant artefacts via template switching.

The novel finding of a genetic bottleneck occurring in the *acute* phase in all subjects regardless of outcome, calls for comprehensive investigation of the determinants of this phenomenon to identify key factors driving HCV evolution towards chronicity. Consistent with the results of this study, an early reduction in viral diversity within the Envelope region was observed during the *acute* phase in subjects who subsequently resolved primary HCV infection [19]. This result is somewhat in contrast with the patterns of viral diversity reported by Wang and colleagues who analysed the Envelope region using NGS in three subjects with symptomatic HCV infection [30]. These authors concluded that there was limited diversification in the early phase of infection, which then increased during the chronic phase. However, this conclusion was derived from analysis of a limited region of the genome, and with only one subject sampled longitudinally in the *acute* phase (assuming a median interval of 50 days between infection and symptom onset). Also, quantification of viral variants was performed only on two short segments of 200 nt and with limited NGS coverage.

The sequential bottleneck events followed by selective sweeps reported here highlights the complex population structure of HCV, and the significance of evolutionary dynamics occurring early in the infection, namely in the first few weeks post-infection. A thorough analysis of these dynamics will ultimately guide understanding of the mechanisms driving establishment of chronic infection. The within-host evolution of HCV observed here is consistent with at least two evolutionary models. Firstly, the observed low effective population size may be the result of strong constraints on infectivity, where only a few viruses can establish infection at each generation. Alternatively, the observed temporal changes in genetic diversity are consistent with a model of evolution via sequential selective sweeps where strong immune pressures drive the establishment of a few escape variants, which then dominate the viral population surviving the bottleneck event. This model has been proposed to explain the epidemiological dynamics occurring in influenza infections [17].

The rapid fixations observed in this study suggest the presence of selective pressures acting on the virus in early infection. The mean number of generations required for a fixation to occur in a neutrally-evolving haploid population (as in RNA viruses) is twice the effective population size, 2 N_eff_ (55). In the case of HCV, using an approximate estimate of N_eff_ = 2000 derived from this study, and a generation time of (say) four days, the estimated fixation time is of the order of years. One explanation for the high fixation rate is that escape from the immune system is likely to be a major driver for such mutations, as well as the occurrence of compensatory mutations. However, the fact that a second genetic bottleneck occurred in the *acute phase* of the infection suggests that chronic infection may not only be the result of escape from host immune responses, but stochastic events may also be relevant, such as genetic drift. In this scenario, strains surviving the second bottleneck may not be those that escaped the immune response, but merely random survivors [reviewed in 16].

A striking finding in the data presented here is that despite the high mutation rate of HCV [8], the large number of infected hepatocytes [61], [62], and the rapid turnover of virions [9], the observed number of substitutions, and of viral variants, during early infection was relatively small, with the majority of substitutions occurring at frequencies below 2%. In the present study up to 60 variants were observed in the HCV Envelope from viral samples at the first available timepoint (Table S3) and up to 100 variants from *pre-chronic* samples. These estimates are consistent with those of Wang and colleagues, however, these authors reported only up to four variants in the *acute* phase, and up to 100 variants from viruses sampled 200 weeks post-infection [30]. It is therefore conceivable that there may be a larger population of variants hidden below the detection threshold in this analysis (0.1%). Studies of HIV diversity have shown that up to 50 amino acid variants are detected in analyses of small epitope regions [54], and at least 15–20 variants have been detected across the full genome [22]. Given that HCV mutates faster than HIV [8], these data raise the question as to why the observed diversity in early HCV is comparable to, or less than, the diversity measured in HIV. Two explanations are possible: firstly, HCV may have an intrinsically higher fitness cost associated with mutations – in another RNA virus, vesicular stomatitis virus, approximately 40% of mutations have been reported to be associated with extinction [8]. One potential instance of relevance identified in the dataset reported here, was in subject 240_Ch, where a CTL epitope in NS3 (surrounding residue 399T) mutated towards I with increasing frequency over the first two timepoints before disappearing below detection. Mutations at this site have been reported to affect viral production [63] (Table S4). A second potential explanation is that early HCV diversity is significantly higher than in subjects with early HIV, but the prevalence of the majority of variants is below the detection threshold – such a skewed distribution has been reported for foot-and-mouth disease virus using NGS [33].

In conclusion, this analysis suggests that the early phase of HCV infection is characterized by strong sequential bottleneck events where diversity is markedly reduced. Whether this is due to strong selective pressure from the host immune response and/or viral fitness cost, remains to be experimentally determined. However, these data suggest that strong selective pressures limit viral evolution early in infection both in subjects that clear infection and those that progress to chronicity. Ultimately, better-informed strategies to modify these selective pressures may alter this outcome, potentially including immunotherapeutic approaches via vaccination, or antiviral therapies to constrain viral replication and hence escape.

## Materials and Methods

### HCV cohort

Four early incident cases were recruited from HITS, which is a prospective cohort of HCV seronegative and HCV RNA negative prison inmates in New South Wales, Australia [42]. Blood samples were collected frequently over 24 weeks following initial viremia, with at least one pre-seroconversion sample collected for each subject (Table S1). HCV antibody testing was performed as described [42]. The date of infection was estimated by subtracting the average pre-seronversion window period, which has been estimated at 51 days [64]–[66], from the midpoint between last seronegative and first seropositive timepoints.

HCV antibody testing was performed using the qualitative Abbott ARCHITECT anti-HCV chemiluminescent microparticle immunoassay (Abbott Diagnostics, Abbott Park, IL, USA). Qualitative HCV RNA detection was performed either using the VERSANT HCV RNA Qualitative Transcription Mediated Amplification (TMA) assay (Bayer Diagnostics, Emeryville, CA, USA; lower limit of detection: 3,200 copies/ml) or COBAS AmpliPrep/COBAS TaqMan HCV assay (Roche, Branchburg, NJ, USA; lower limit of detection 223 genome copies/ml).

### Ethics statement

Ethical approvals were obtained from Human Research Ethics Committees of Justice Health (reference number GEN 31/05), New South Wales Department of Corrective Services (reference number 05/0884), and the University of New South Wales (reference numbers 05094, 08081), all located in Sydney, Australia. Written informed consent was obtained from the participants.

### RNA extraction and amplification of partial 5′UTR to 3′ NS5B for NGS

Viral RNA was extracted as previously described [67]. Near full length HCV cDNA was synthesized from 8 µl of viral RNA with the Superscript III First-Strand Synthesis System for RT-PCR (Invitrogen, Mt. Waverley, Australia) and a genotype specific primer (Table S6) according to manufacturers' instructions. The region spanning nt 98 to 9314 (nt position designated according to H77, GenBank accession number AF011751) was amplified from the cDNA in two (5′ UTR - 3′ NS2 and 3′ NS2 - 3′ NS5B) or three fragments (5′ UTR -3′ NS2, 3′ NS2 - 5′ NS4B, and 5′ NS4B - 3′ NS5B) with genotype specific primers (Table S6) in a single PCR round with SequalPrep Long PCR kit (Invitrogen). First round PCRs were performed using 5 µl of cDNA added to 15 µl of PCR reaction mix according to manufacturers' instructions (Invitrogen). First round PCR was carried out for 94°C for 2 min, then 10 cycles of 94°C, 50°C, 68°C for 30 sec, 30 sec and 6 min, respectively and followed with an additional 25 cycles of 94°C, 58°C, 52°C for 30 sec, 30 sec and 6 min (plus 20 sec extra each cycle), respectively. Second round PCR was performed using 5 µl of first round product and an inner set of genotype specific primers (Table S6) added to 45 µl of PCR reaction mix. Reaction mix and conditions were as described above with one exception; the annealing temperature was raised to 60°C.

For the GT3, and some GT1a samples (subject 686 2/3/09, 23/3/09 and 6/4/09, and subject 23 2/6/09, 2/7/09 and 1/9/09), it was not possible to obtain a single amplicon spanning the 3′ NS2 to 3′ NS5B and this region was amplified from the first round product in two fragments, 3′ NS2 to 5′ NS4B and 5′ NS4B to 3′ NS5B. Nested round amplification was performed with Platinum *Taq* High Fidelity (Invitrogen). Reaction conditions were as specified in the manufacturers' instructions with 5 µl of first round product added as template.

PCR products were electrophoresed on 0.8% agarose in 0.5× TBE buffer and products of the correct size were gel purified with the QIAquick gel extraction kit (Qiagen) and confirmed to be the amplicon of interest by direct sequencing on an ABI 3730 DNA Analyzer (Applied Biosystems, Foster City, CA, US) using dye-terminator chemistry.

### Roche 454 FLX sequencing

PCR amplicons were quantified with the Picogreen dsDNA assay (Invitrogen). Genome fragments amplified from the same timepoint were pooled in equimolar amounts to obtain equal coverage, and submitted for library preparation before subsequent NGS [23] using 454 Roche FLX Titanium at the Murdoch University, Perth, Australia. Pyrosequencing was performed in three independent runs. The first two runs were carried out without bar-coding. The last run was performed using bar-coding given the improvement of this technology over the course of the study. The last run included two samples for each of the subjects 23_Ch, 686_Cl, and 360_Cl. All other samples were analyzed in the first two runs.

### E1/E2 clones

The 3′ end of Core to 5′ end of p7 region was amplified for selected timepoints from each subject with genotype specific primers (Table S6) and Platinum *Taq* DNA polymerase High Fidelity, using the gel purified PCR product submitted for deep sequencing as the DNA template. The 2534 bp product was TA ligated into the pGEM-T Easy vector (Promega, Wisconsin, United States). Individual colonies were screened by PCR for inserts of the appropriate size with vector specific primers and the amplicons sequenced.

### 454 FLX data alignment

454 FLX sequence reads were removed prior to the assembly stage if they were: shorter than 55 bp and had an average quality score <20. The terminal 20 nt were removed from all remaining reads. These remaining sequences were aligned with a nucleotide identity threshold of 95% against each subject's unique consensus sequence with the alignment tool, MOSAIK (http://bioinformatics.bc.edu/marthlab/Mosaik). The recommended parameters for 454 data were used in aligning these reads. Each subject's consensus sequence was derived from sequencing the gel purified PCR products on the ABI 3730 DNA Analyzer spanning the near full-length genome generated at the first viremic timepoint. The quality of the aligned file was assessed and reads were excluded from the alignment on the following basis: i) hypermutated sequence (sequences with more than 5 nt mutations within the first 20 position of the read in either of the ends; ii) reads with indels that resulted in a frame-shift or reads with a high frequency (5%) of indels relative to the reference.

### SNP detection and haplotype reconstruction

Aligned 454 reads were further analyzed with a Bayesian probabilistic method implemented in the software package ShoRAH [68], [69]. This software was used to: i) perform error correction on 454 FLX reads; ii) estimate the distribution of SNPs and their prevalence, using a detection threshold of 0.1%; iii) reconstruct HCV variants (haplotypes) in local windows of 400 nt; iv) reconstruct HCV haplotypes on the Envelope region of the HCV genome (∼1000 nt in length); and v) to estimate the frequency of occurrence of reconstructed variants within the sample. The result of this global analysis on the Envelope region was compared to the available sequences obtained from standard cloning and sequencing. The ShoRAH analyses were performed in triplicate for each dataset to ensure that the stochastic nature of Bayesian statistics based on Monte Carlo Markov Chain simulations was not affecting the results. Only SNPs and variants detected in all the three simulation runs were considered for further analyses.

Further automated cleaning of the SNPs generated via the ShoRAH method was performed, based on the following criteria: (i) sequences where the reverse and forward strand differed in a frequent base substitution, allowing only a difference of 10% between forward and reverse reads; (ii) SNPs which were only present at the end of reads; and (iii) if the nucleotides adjacent to the SNP were part of a homopolymeric stretch. The results from ShoRAH were also compared to those obtained using a cut-off method, such as that provided in the software package, Varscan [70].

For the analysis of the Envelope region of the genome, only reconstructed variants with frequencies >2.5% were considered for further analysis (see Supporting Text S1). The probabilistic nature of the clustering algorithm allows for estimation of the reliability of predictions. In the Bayesian scenario implemented in ShoRAH, the fraction of iterations in which a haplotype is reported estimates the posterior probability of the existence of that haplotype. This posterior probability provides a confidence level for the haplotype. Only haplotypes with confidence values (posterior probabilities) greater than 0.9 are reported. Scripts and parameter values used for the analyses are available upon request. Parameters of ShoRAH have been chosen through a detailed preliminary analysis on the sensitivity of these parameters in collaboration with Dr Zagordi (personal communication).

### Founder virus analysis

PoissonFitter was used to test the hypothesis that a single virus establishes infection [71]. PoissonFitter performs two tests: one test is based on the fit of the Poisson model to the frequency distribution of the Hamming distance observed in each sample; the other is a topological test to verify that observed frequencies are distributed according to a star-like phylogeny (for this test, no formal statistic is available and consequently no p-value is obtained). In this model the main assumption is that a single founder virus evolves under neutral evolution, generating a star-like phylogeny, with a distribution of mutations conforming to a Poisson distribution [15], [45]. This means that early selective pressures compromise the statistical analysis. For this reason, only HCV sequences obtained from the first available viremic time point for each subject were included in the founder virus analysis. PoissonFitter test was performed on reconstructed viral variants in E1/E2 region of the genome, as well as on reconstructed variants obtained from 400 nt windows sliding across the full genome. A type 1 error threshold α = 0.01 was assumed to conclude whether the Poisson test was rejected. For the initial analysis, in each window a conclusion was made whether one, or more than one, founder virus explained the observations. In cases where the two tests described above were discordant (i.e. a significant fit with Poisson test of a single founder virus, but no star-like phylogeny – which occurred in seven analyses; or the Poisson test suggested more than one founder while the topological test conformed to a star-like phylogeny – which occurred in eight analyses) then early stochastic events were assumed to have occurred, which limited the validity of the tests. These early events could result from an early selective pressure, or strong fitness advantage associated with early mutations. In this analysis, the mutation rate for the HCV genome was set to 1.2×10^−4^, as recently estimated [72]. We assumed the same value was constant across the HCV genome.

### Phylogenetic analysis

Sequences from standard cloning and reconstructed haplotypes were visualized and curated with MEGA [73] and R packages. Phylogenetic and evolutionary analyses were performed with PhyML [74]. Trees were constructed from sequences using a GTR substitution model with gamma invariant sites, as suggested by analyses using ModelTEST [75]. Trees were visualized with FigTree. Estimates of tMRCA were performed with PoissonFitter and with BEAST [76].

Demographic reconstruction of the within-subject viral population was performed with skyline plots implemented in BEAST [47], [63]. The analysis was performed using both a strict clock model and a relaxed molecular clock model. The length of the MCMC chain was chosen so that the effective sample size (ESS) for each parameter was > 100. Bayesian factors were then used to decide the best model. For each subject, the null hypothesis of a strict clock was not rejected according to the Bayes Factor calculated from the posterior distributions obtained from each model (BF<2).

The evolutionary distances were estimated as within-group mean genetic distances estimated using Maximum Composite Likelihood (implemented in Mega 5) from reconstructed variants from NGS in the E1/E2 region of the HCV genome.

### CD8+ epitope predictions

Subjects were HLA typed (Table S2) and MHC Class I restricted epitopes were predicted using available algorithms in http://www.immuneepitope.org. For each subject, several different algorithms were tested with similar results. For simplicity, the results presented are derived from prediction with NetMHC http://www.cbs.dtu.dk/services/NetMHC/, which uses an artificial neural network for predictions. Known HCV epitopes were retrieved from the database http://www.immuneepitope.org (last access, 24 January 2011).

Predicted HLA-A or B restricted epitopes derived from consensus sequence of the viral genome at each time point and in each subject, were used to test the hypothesis that the distribution of Shannon entropy measures in genomic regions carrying predicted epitopes differ from the distribution of Shannon entropy values in region without predicted epitopes. Statistical testing was performed using the Mann-Whitney U test. A one-tailed test was performed to examine the hypothesis that epitope regions had higher SE measures than non-epitope regions.

## Supporting Information

Figure S1 **Founder virus analysis based on E1/HVR1 of the viral genome.** Panels A and B show the analyses for two subjects who developed chronic infection (240_Ch, 23_Ch) followed from pre-seroconversion timepoints. Panels C and D show the analysis for two subjects who cleared the infection (360_Cl, 686_Cl). Phylogenetic reconstructions and highlighter plots are shown, illustrating the genetic relatedness between HCV variant sequences. Names of each sequence are labeled with a letter (H for haplotype, and C for clone), with the first number representing the sampling timepoint and with the second number representing either the prevalence of the haplotype or the clone number. The phylogenetic trees of subjects 686_Cl, 360_Cl and 240_Ch (panels A, C, D) are consistent with an infection arising from a single founder. The fit with a Poisson model is also consistent with a single founder (p-value > 0.1, see text). As shown by the highlighter plots, founder viruses are identified as the consensus sequence and coincided with the most prevalent variant reconstructed from NGS data, (e.g. for subject 686_Cl H1_0.70 is identical to the consensus sequence and to six clone sequences, see Highlighter plot). The highlighter plots also show the random distribution of mutated sites with respect to the founder sequence (master), which is consistent with a star-like phylogeny. The phylogenetic analysis in 23_Ch (panel B) is consistent with an infection originated from two founder viruses (indicated with an asterisk in the highlighter plot) giving rise to two major clusters, 23A_F_ and 23B_F_. This is consistent with the rejection of the Poisson model (p-value = 0). Phylogenetic trees were obtained using PhyML, with Maximum Likelihood methods using a GTR model of substitution as suggested by model testing.
(TIF)

Figure S2 **Fit of the distribution of Hamming distances with a Poisson model performed on the Envelope region, specifically on partial E2 (932 nt).** Each panel shows a subject; the red line is the fit of the distribution of Hamming distances (histogram) calculated from sequences derived from the reconstruction of NGS data and from standard cloning. The poor fit outcome for subject 23_Ch indicated the presence of more than one founder virus. See also Table S3.
(TIF)

Figure S3 **Evolutionary dynamics of HCV variants over the E1/HVR1 region of the genome.** Sequence analyses of the two subjects who developed chronic infection, 240_Ch (A), and 23_Ch (B) revealed the presence of selective sweeps. These sweeps led to the emergence of new variants that replaced the founder viruses (identified with an asterisk). Phylogenetic trees (left panels) display nucleotide sequences of reconstructed haplotypes derived from NGS data and clonal sequences. Names of each sequence are labeled with a letter (H for haplotype and C for clone), with the first number representing the sampling timepoint and with the second number representing either the prevalence of the haplotype or the clone number. Colors are also used to portray the sampling timepoint (see legend). Infection dynamics for subject 240_Ch are consistent with a single founder, identified with the most prevalent strain of cluster 240A (H2_0.65, indicated with an asterisk), and with clones C2_6, and C2_17, and with the consensus of the sequences from time-point 1. The *pre-chronic* phase (corresponding with the color-coded time ranges 5 and 6) of infection shows the emergence and dominance of a new subgroup of viruses. 23_Ch has two founder viruses that successfully initiated the infection (H1_0.25 and H1_0.27, indicated with an asterisk). A new cluster emerged from the founder cluster and became dominant in the *pre-chronic* phase. Trees are calculated using Maximum Likelihood method (implemented in PhyML).
(TIF)

Figure S4 **Evolutionary dynamics of HCV variants over the partial NS3 region of the genome.** Sequence analyses (over 400 nt window) of the two subjects who developed chronic infection, 240_Ch (panel A), and 23_Ch (panel B) revealed the presence of selective sweeps. These sweeps led to the emergence of new variants that replaced the founder virus(es). Phylogenetic trees display nucleotide sequences of reconstructed haplotypes derived from NGS data and clonal sequences. Names of each sequence are labeled with a letter (H for haplotype), with the first number representing the sampling timepoint and with the second number representing the prevalence of the haplotype. Colors are also used to portray the sampling timepoint (see legend).
(TIF)

Figure S5 **Demographic reconstruction of the viral populations.** Demographic reconstruction from E1-HVR1 and E2 sequences for subjects who cleared the infection (686_Cl and 360_Cl). In both subjects and in both genomic regions the estimated effective population size (Nτ, the product of the effective population size and generation length in days) is higher than in chronic subjects with peak around 10^4^. Subject 686_Cl shows a moderate increase in viral diversity over time, corroborating resuts from Shannon entropy and Poisson-Fitter tests.
(TIF)

Table S1 Demographic and laboratory characteristics of the subjects.
(DOC)

Table S2 Summary of the next generation sequencing (NGS) data obtained for each subject.
(DOC)

Table S3 PoissonFitter test results for the single founder virus analysis for each subject.
(DOC)

Table S4 Phenotypic analyses of the non-synonymous substitutions over the course of the infection.
(XLS)

Table S5 Analysis of amino acid substitutions within predicted HLA restricted cytotoxic T cell epitopes.
(DOC)

Table S6 Primers used to amplify viral sequences.
(DOC)

Text S1 **Haplotype reconstruction and method validation.** Detailed information on the haplotype reconstruction from NGS data. This methodology was validated with a 454 FLX run performed in duplicate on four unique plasmids with known sites of variation.
(DOC)
