# Supplementary figures and images for: Sequential Bottlenecks Drive Viral Evolution in Early Acute Hepatitis C Virus Infection
Source: PLoS Pathog. 2011 Sep 1;7(9):e1002243. doi: 10.1371/journal.ppat.1002243 (PMC3164670; doi:10.1371/journal.ppat.1002243)

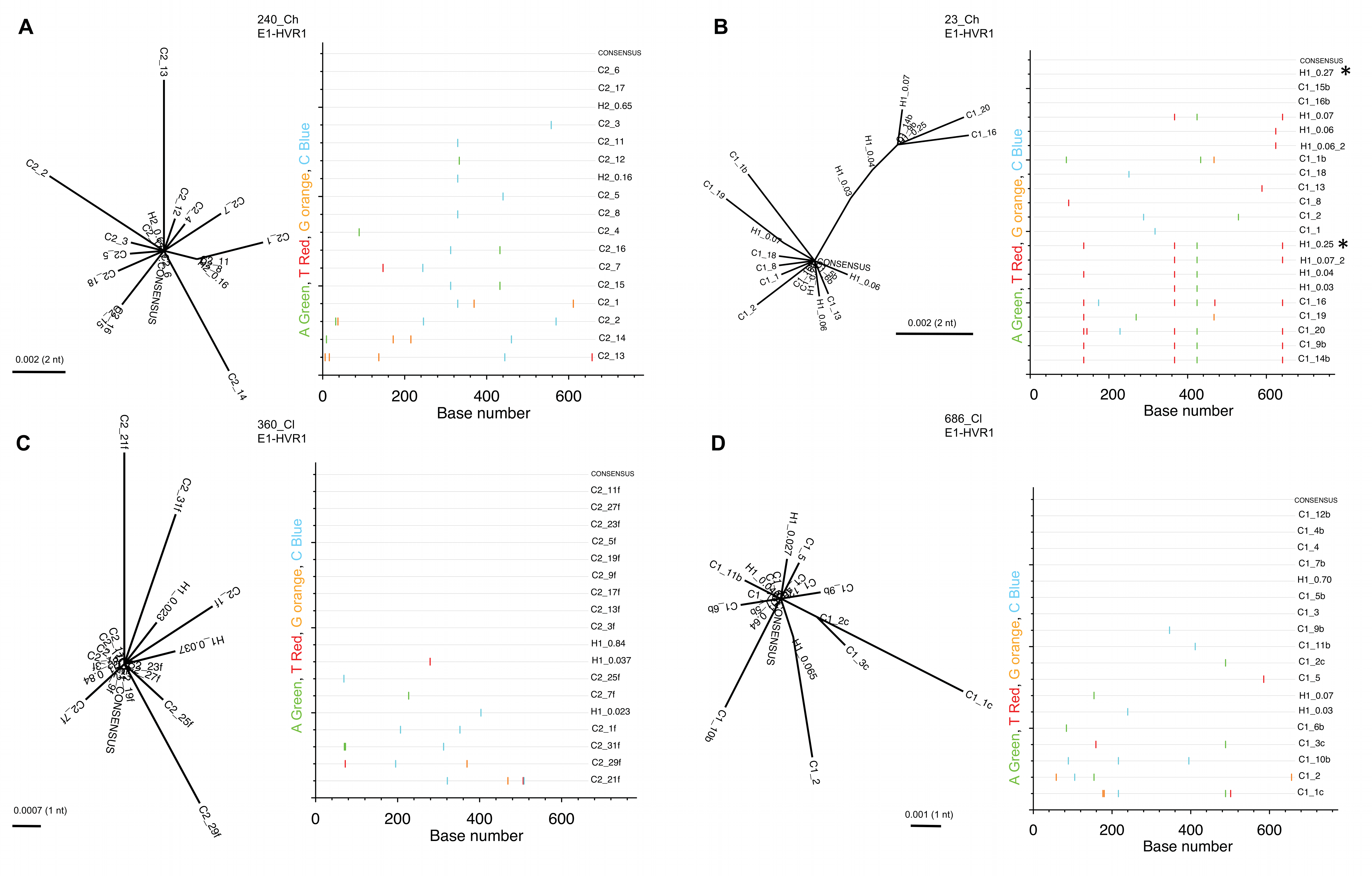

Supplement: Figure S1 — Founder virus analysis based on E1/HVR1 of the viral genome. Panels A and B show the analyses for two subjects who developed chronic infection (240_Ch, 23_Ch) followed from pre-seroconversion timepoints. Panels C and D show the analysis for two subjects who cleared the infection (360_Cl, 686_Cl). Phylogenetic reconstructions and highlighter plots are shown, illustrating the genetic relatedness between HCV variant sequences. Names of each sequence are labeled with a letter (H for haplotype, and C for clone), with the first number representing the sampling timepoint and with the second number representing either the prevalence of the haplotype or the clone number. The phylogenetic trees of subjects 686_Cl, 360_Cl and 240_Ch (panels A, C, D) are consistent with an infection arising from a single founder. The fit with a Poisson model is also consistent with a single founder (p-value > 0.1, see text). As shown by the highlighter plots, founder viruses are identified as the consensus sequence and coincided with the most prevalent variant reconstructed from NGS data, (e.g. for subject 686_Cl H1_0.70 is identical to the consensus sequence and to six clone sequences, see Highlighter plot). The highlighter plots also show the random distribution of mutated sites with respect to the founder sequence (master), which is consistent with a star-like phylogeny. The phylogenetic analysis in 23_Ch (panel B) is consistent with an infection originated from two founder viruses (indicated with an asterisk in the highlighter plot) giving rise to two major clusters, 23AF and 23BF. This is consistent with the rejection of the Poisson model (p-value = 0). Phylogenetic trees were obtained using PhyML, with Maximum Likelihood methods using a GTR model of substitution as suggested by model testing. (TIF) [file ppat.1002243.s001.tif]

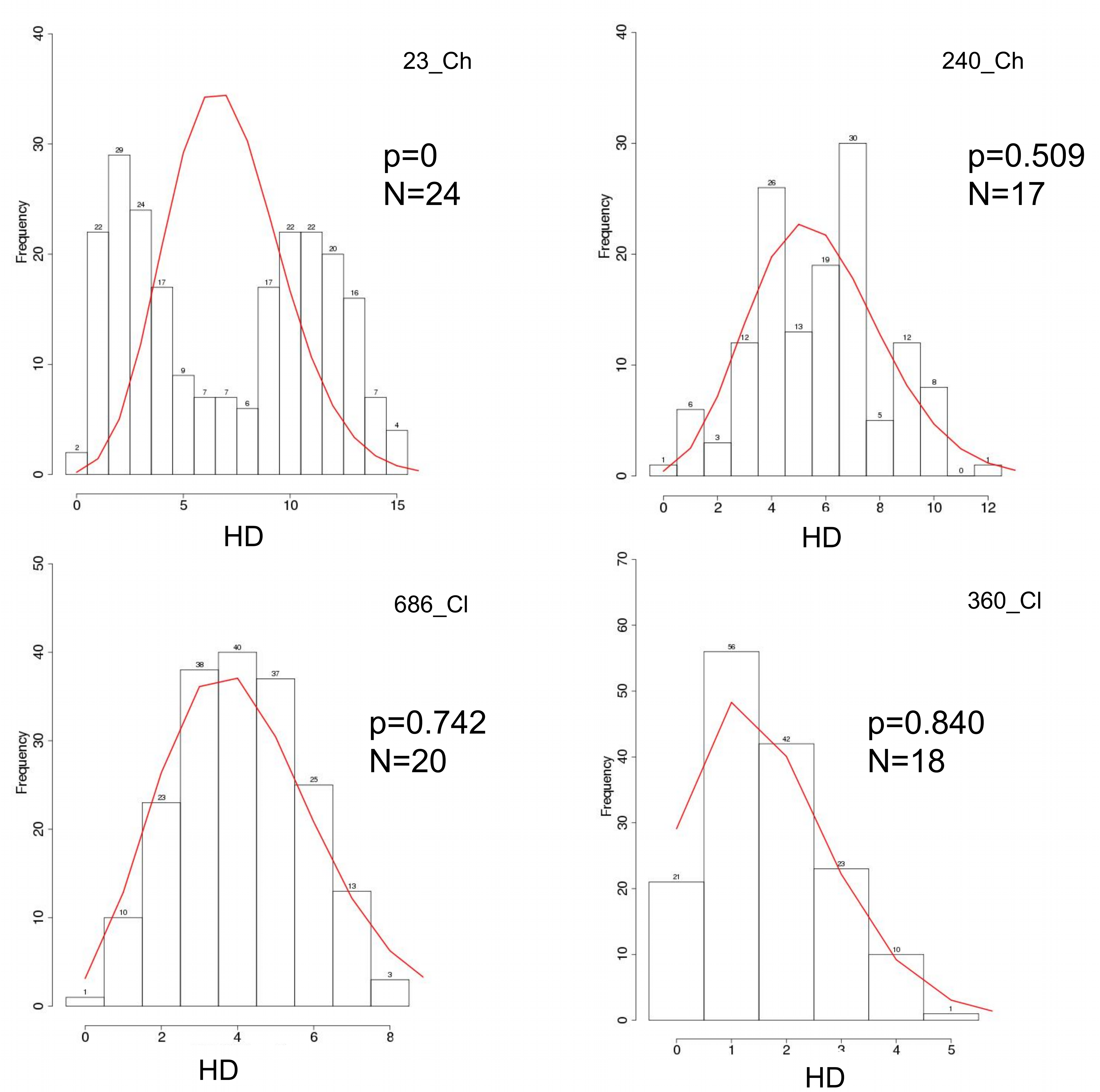

Supplement: Figure S2 — Fit of the distribution of Hamming distances with a Poisson model performed on the Envelope region, specifically on partial E2 (932 nt). Each panel shows a subject; the red line is the fit of the distribution of Hamming distances (histogram) calculated from sequences derived from the reconstruction of NGS data and from standard cloning. The poor fit outcome for subject 23_Ch indicated the presence of more than one founder virus. See also Table S3. (TIF) [file ppat.1002243.s002.tif]

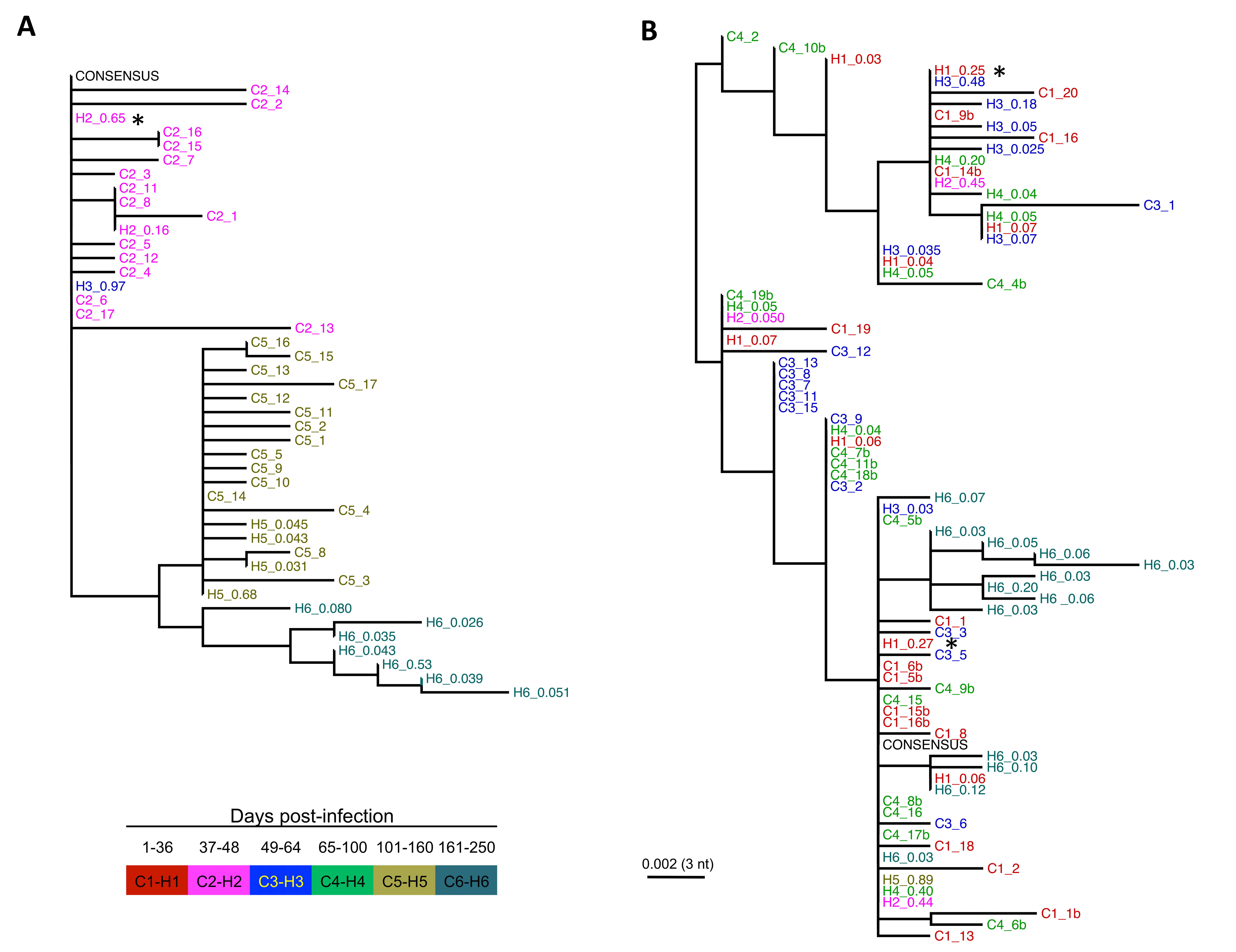

Supplement: Figure S3 — Evolutionary dynamics of HCV variants over the E1/HVR1 region of the genome. Sequence analyses of the two subjects who developed chronic infection, 240_Ch (A), and 23_Ch (B) revealed the presence of selective sweeps. These sweeps led to the emergence of new variants that replaced the founder viruses (identified with an asterisk). Phylogenetic trees (left panels) display nucleotide sequences of reconstructed haplotypes derived from NGS data and clonal sequences. Names of each sequence are labeled with a letter (H for haplotype and C for clone), with the first number representing the sampling timepoint and with the second number representing either the prevalence of the haplotype or the clone number. Colors are also used to portray the sampling timepoint (see legend). Infection dynamics for subject 240_Ch are consistent with a single founder, identified with the most prevalent strain of cluster 240A (H2_0.65, indicated with an asterisk), and with clones C2_6, and C2_17, and with the consensus of the sequences from time-point 1. The pre-chronic phase (corresponding with the color-coded time ranges 5 and 6) of infection shows the emergence and dominance of a new subgroup of viruses. 23_Ch has two founder viruses that successfully initiated the infection (H1_0.25 and H1_0.27, indicated with an asterisk). A new cluster emerged from the founder cluster and became dominant in the pre-chronic phase. Trees are calculated using Maximum Likelihood method (implemented in PhyML). (TIF) [file ppat.1002243.s003.tif]

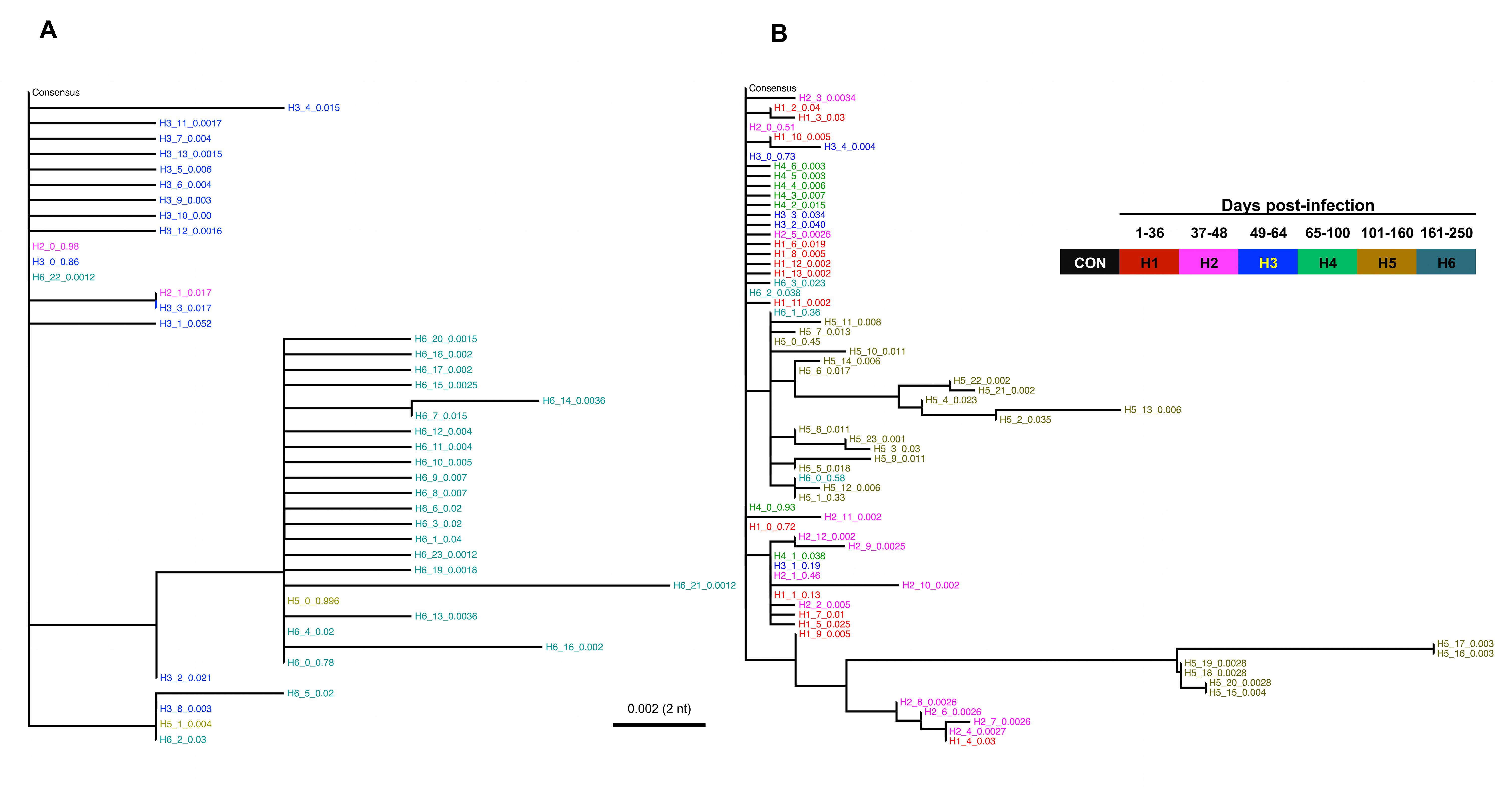

Supplement: Figure S4 — Evolutionary dynamics of HCV variants over the partial NS3 region of the genome. Sequence analyses (over 400 nt window) of the two subjects who developed chronic infection, 240_Ch (panel A), and 23_Ch (panel B) revealed the presence of selective sweeps. These sweeps led to the emergence of new variants that replaced the founder virus(es). Phylogenetic trees display nucleotide sequences of reconstructed haplotypes derived from NGS data and clonal sequences. Names of each sequence are labeled with a letter (H for haplotype), with the first number representing the sampling timepoint and with the second number representing the prevalence of the haplotype. Colors are also used to portray the sampling timepoint (see legend). (TIF) [file ppat.1002243.s004.tif]

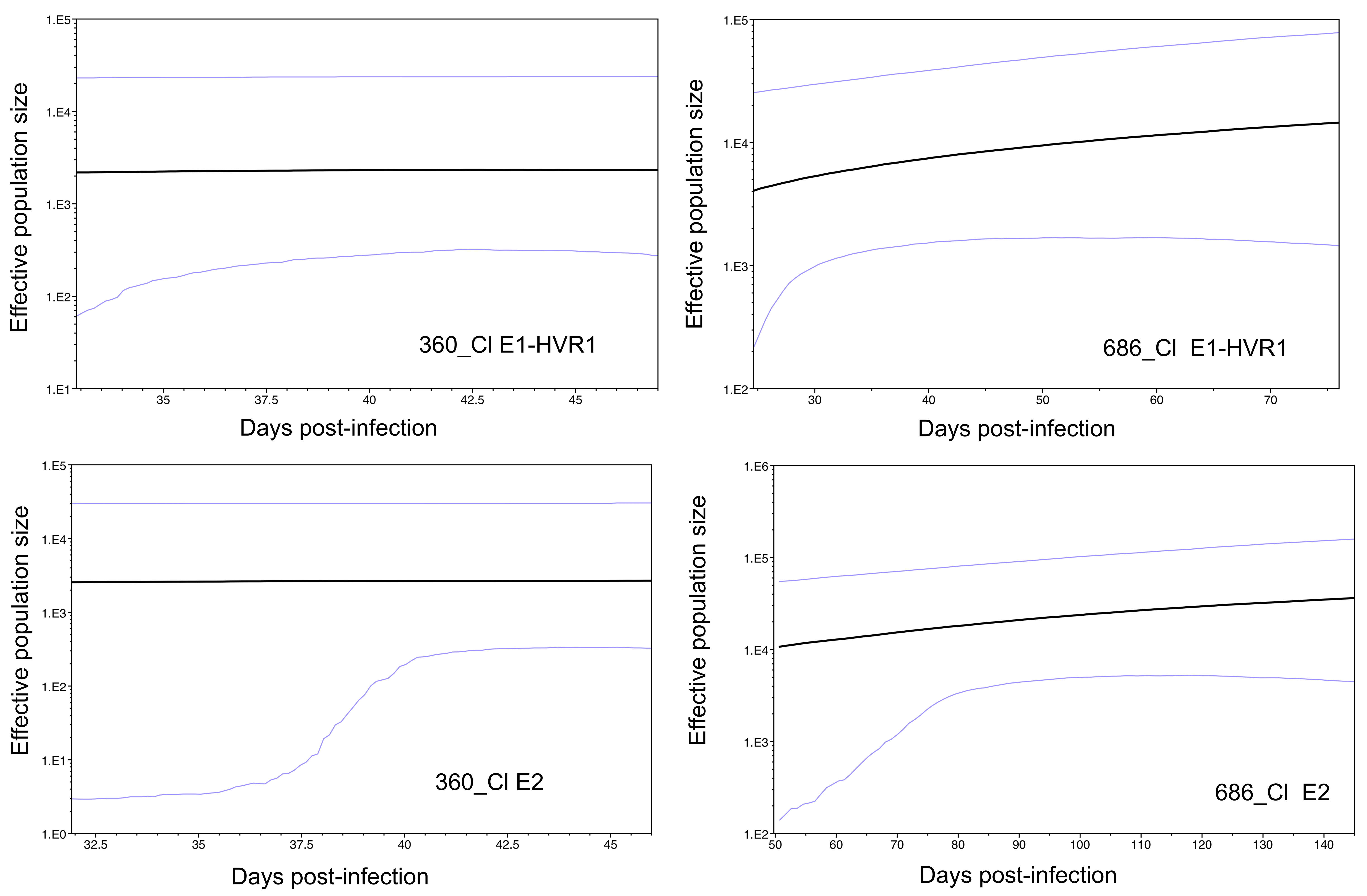

Supplement: Figure S5 — Demographic reconstruction of the viral populations. Demographic reconstruction from E1-HVR1 and E2 sequences for subjects who cleared the infection (686_Cl and 360_Cl). In both subjects and in both genomic regions the estimated effective population size (Nτ, the product of the effective population size and generation length in days) is higher than in chronic subjects with peak around 104. Subject 686_Cl shows a moderate increase in viral diversity over time, corroborating resuts from Shannon entropy and Poisson-Fitter tests. (TIF) [file ppat.1002243.s005.tif]
