# Supplementary material for: Sequential Bottlenecks Drive Viral Evolution in Early Acute Hepatitis C Virus Infection
Source: PLoS Pathog. 2011 Sep 1;7(9):e1002243. doi: 10.1371/journal.ppat.1002243 (PMC3164670; doi:10.1371/journal.ppat.1002243)
Supplement: Table S1 — Demographic and laboratory characteristics of the subjects. (DOC) [file ppat.1002243.s006.doc]

**Table S1**. **Demographic and laboratory characteristics of the subjects**

| **ID** | **Sex** | **Disease**  **outcome** | **GT** | **HLA-I**  **(A, B)** | **HLA-II (DRB1)** | **Estimated days post infection (DPI)** | **HCV Ab** | **HCV RNA** | **RNA level (IU/mL)** |
| --- | --- | --- | --- | --- | --- | --- | --- | --- | --- |
| 240_Ch | M | Chronic | 3a | A0201 | 0101 | -61 | - | - | 0 |
|  |  |  |  | B1501 | 0701 | 44 | - | + | 54,887 |
|  |  |  |  | B5701 |  | 57 | + | + | 85,473 |
|  |  |  |  |  |  | 71 | + | + | 64,063 |
|  |  |  |  |  |  | 85 | + | + | 6,051 |
|  |  |  |  |  |  | 99 | + | + | 497 |
|  |  |  |  |  |  | 113 | + | + | 4,862 |
|  |  |  |  |  |  | 140 | + | + | 1,034 |
|  |  |  |  |  |  | 159 | + | + | 44,449 |
|  |  |  |  |  |  | 249 | + | + | 29,552 |
|  |  |  |  |  |  | 477 | + | + | 62,174 |
| 23_Ch | M | Chronic | 1a | A0201 | 0401 | -165 | - | - | 0 |
|  |  |  |  | B4402 | 0701 | 36 | - | + | 19,234,348 |
|  |  |  |  | B5701 |  | 44 | - | + | 17,907,338 |
|  |  |  |  |  |  | 60 | + | + | 8,121,396 |
|  |  |  |  |  |  | 74 | + | + | 397,185 |
|  |  |  |  |  |  | 85 | + | + | 3,218 |
|  |  |  |  |  |  | 102 | + | + | 398 |
|  |  |  |  |  |  | 136 | + | + | 2,843,176 |
|  |  |  |  |  |  | 167 | + | + | 5,896,155 |
|  |  |  |  |  |  | 304 | + | + | 51,6945 |
| 686_Cl | F | Clearer | 1a | A0101 | 0701 | 33 | - | + | 287,770 |
|  |  |  |  | A3001 | 1502 | 40 | - | + | 2,404,901 |
|  |  |  |  | B1302 |  | 61 | + | + | >69,000,000 |
|  |  |  |  | B5201 |  | 75 | + | + | 5,106,563 |
|  |  |  |  |  |  | 89 | + | + | 2,267 |
|  |  |  |  |  |  | 110 | + | + | <15 |
|  |  |  |  |  |  | 117 | + | + | <15 |
|  |  |  |  |  |  | 149 | + | - | 0 |
|  |  |  |  |  |  | 264 | + | - | 0 |
| 360_Cl | F | Clearer | 3a | A3201 | 0301 | -101 | - | - | 0 |
|  |  |  |  | A6801 | 0802 | 30 | - | + | 5,648,631 |
|  |  |  |  | B1402 |  | 44 | - | + | 4,617,483 |
|  |  |  |  | B4402 |  | 58 | + | + | 14,170 |
|  |  |  |  |  |  | 71 | + | + | 15,938 |
|  |  |  |  |  |  | 83 | + | + | 1,060 |
|  |  |  |  |  |  | 97 | + | + | <15 |
|  |  |  |  |  |  | 132 | + | + | 57 |
|  |  |  |  |  |  | 223 | + | - | 0 |
