# Supplementary material for: Sequential Bottlenecks Drive Viral Evolution in Early Acute Hepatitis C Virus Infection
Source: PLoS Pathog. 2011 Sep 1;7(9):e1002243. doi: 10.1371/journal.ppat.1002243 (PMC3164670; doi:10.1371/journal.ppat.1002243)
Supplement: Table S2 — Summary of the next generation sequencing (NGS) data obtained for each subject. (DOC) [file ppat.1002243.s007.doc]

Table S2. Summary of the next generation sequencing (NGS) data obtained for each subject.

| **Subject** | **Date of sample**  **(days post-infection)** | **Matched**  **reads** | **Unmatched**  **reads** | **Average read**  **length (bp)** | **Average**  **coverage** | **Genome length**  **analysed (bp)** |
| --- | --- | --- | --- | --- | --- | --- |
| 240_Ch | 44 | 92580 | 760 | 375 | 3778 | 9226 |
|  | 57 | 83209 | 4285 | 334 | 3024 | 9226 |
|  | 159 | 108740 | 1662 | 356 | 4211 | 9226 |
|  | 249 | 98711 | 1810 | 327 | 3508 | 9226 |
| 23_Ch | 36 | 89687 | 2754 | 367 | 3598 | 9138 |
|  | 44 | 57931 | 3546 | 355 | 2258 | 9138 |
|  | 60 | 50539 | 3151 | 375 | 2070 | 9138 |
|  | 74 | 86831 | 4464 | 367 | 3484 | 9138 |
|  | 136 | 49431 | 3492 | 355 | 2610 | 6500 |
|  | 167 | 75584 | 18305 | 359 | 2742 | 9138 |
| 686_Cl | 33 | 95341 | 1341 | 368 | 3839 | 9172 |
|  | 40 | 60977 | 3590 | 347 | 2316 | 9172 |
|  | 61 | 115479 | 4709 | 352 | 4444 | 9172 |
|  | 75 | 119179 | 1009 | 349 | 4547 | 9172 |
| 360_Cl | 30 | 67064 | 2456 | 356 | 3130 | 5992 |
|  | 44 | 60186 | 1838 | 346 | 2314 | 5992 |
| **Mean:** | | 81866.7 | 7846.5 | 358.1 | 3092.5 |  |
