# Supplementary material for: Sequential Bottlenecks Drive Viral Evolution in Early Acute Hepatitis C Virus Infection
Source: PLoS Pathog. 2011 Sep 1;7(9):e1002243. doi: 10.1371/journal.ppat.1002243 (PMC3164670; doi:10.1371/journal.ppat.1002243)
Supplement: Table S3 — PoissonFitter test results for the single founder virus analysis for each subject. (DOC) [file ppat.1002243.s008.doc]

**Table S3. PoissonFitter test results for the single founder virus analysis for each subject.**

Subject 23_Ch

| **Genomic region** | **No. haplotypes tested^a^** | **Max. HD**^b^ | **Poisson estimated tMRCA**^d^ | **GOF**^c^  **p-value** | **Star-like phylogeny** | **Explanation for deviation from model** | **No. founder viruses^f^** | **λ Poisson** |
| --- | --- | --- | --- | --- | --- | --- | --- | --- |
| E1-HVR1  (665) | 24 | 15 | 36 (29, 44)  30 (11, 60) ^e^ | 0.0362 | No | >1 | 2 | 0.3231 |
| E2  (875) | 24 | 15 | 54 (44, 64)  63 (28, 119) ^e^ | 0.0 | No | >1 | 2 | 0.578 |
| ***Analysis on reconstructed HCV variants across the genome in windows of 400 nucleotides*** | | | | | | | | |
| 1-400 | 8 (2830) | 3 | 33 (16, 49) | 0.597 | No | Early stochastic | 1 | 0.461 |
| 400-800 | 12 (2930) | 2 | 31 (23, 39) | 0.041 | Yes |  | 1 | 0.217 |
| 800-1200 | 15 (2830) | 5 | 44 (37, 52) | 0.023 | No | >1 | 2 | 0.20 |
| 1200-1600 | 20 (2830) | 10 | 44 (41, 48) | <10^-10^ | No | >1 | 2 | 0.20 |
| 1600-2000 | 13 (2902) | 10 | 85 (65, 105) | <10^-10^ | No | >1 | 2 | 0.55 |
| 2000-2400 | 15 (2882) | 7 | 59 (48, 71) | 0.310 | No | Early stochastic | 2 | 3.30 |
| 2400-2800 | 12 (1950) | 6 | 41 (26, 56) | 0.830 | Yes |  | 1 | 0.36 |
| 2800-3200 | 15 (2141) | 5 | 51 (41, 60) | 0.043 | No | Early stochastic | 1 | 0.24 |
| 3200-3600 | 13 (2139) | 3 | 57 (38, 76) | 0.050 | No |  | 1 | 0.21 |
| 3600-4000 | 9 (2475) | 8 | 56 (41, 71) | 0.722 | Yes |  | 1 | 0.87 |
| 4000-4400 | 15 (2675) | 10 | 63 (39, 86) | 0.009 | No | >1 | 2 | 0.61 |
| 4400-4800 | 23 (2883) | 8 | 56 (23, 88) | <10^-5^ | No | >1 | 2 | 2.29 |
| 4800-5200 | 13 (2007) | 3 | 47 (34, 60) | 0.466 | No |  | 1 | 0.36 |
| 5200-5600 | 13 (2007) | 6 | 53 (43, 63) | 0.774 | No | Early stochastic | 1 | 0.16 |
| 5600-6000 | 7 (1950) | 6 | 19 (9, 28) | 0.389 | Yes |  | 1 | 0.389 |
| 6000-6400 | 11 (2233) | 7 | 39 (35, 43) | 0.438 | Yes |  | 1 | 2.17 |
| 6400-6800 | 11 (2515) | 5 | 43 (32, 55) | 0.263 | Yes |  | 1 | 0.33 |
| 6800-7200 | 5 (2345) | 2 | 20 (17, 22) | 0.173 | Yes |  | 1 | 0.33 |
| 7200-7600 | 15 (2849) | 8 | 50 (40, 61) | 0.872 | No | Early stochastic | 1 | 0.37 |
| 7600-8000 | 11 (2521) | 5 | 37 (30, 43) | 0.046 | Yes | >1 | 1 | 2.00 |
| 8000-8400 | 10 (2737) | 4 | 40 (31, 49) | 0.298 | Yes |  | 1 | 2.20 |
| 8400-8800 | 12 (2743) | 4 | 36 (24, 48) | 0.845 | Yes |  | 1 | 0.26 |
| 8800-9200 | 7 (2297) | 3 | 23 (12, 34) | 0.516 | Yes | Early stochastic | 1 | 0.24 |

Subject 240_Ch

| **Genomic region** | **No. haplotypes tested^a^** | **Max. HD**^b^ | **Poisson estimated tMRCA**^d^ | **GOF**^c^  **p-value** | **Star-like phylogeny** | **Explanation for deviation from model** | **No. founder viruses^f^** | **λ Poisson** |
| --- | --- | --- | --- | --- | --- | --- | --- | --- |
| E1-HVR1  (670) | 18 | 9 | 36 (25, 48)  23 (12,37) ^e^ | 0.5 | Yes |  | 1 | 0.5012 |
| E2  (932 nt) | 17 | 12 | 47 (37, 57)  19 (11,27) ^e^ | 0.509 | Yes |  | 1 | 0.6305 |
| ***Analysis on reconstructed HCV variants across the genome in windows of 400 nucleotides*** | | | | | | | | |
| 1-400 | 25 (4387) | 6 | 23 (19, 26) | 0.068 | Yes |  | 1 | 1.19 |
| 400-800 | 10 (4380) | 3 | 38 (29, 47) | 0.039 | Yes |  | 1 | 0.25 |
| 800-1200 | 25 (4065) | 3 | 38 (36, 40) | <10^-9^ | Yes | Early stochastic | 1 | 0.07 |
| 1200-1600 | 31 (4335) | 5 | 38 (36, 41) | 0.558 | Yes |  | 1 | 0.11 |
| 1600-2000 | 21 (2561) | 7 | 41 (37, 45) | 0.013 | Yes |  | 1 | 2.78 |
| 2000-2400 | 17 (2553) | 4 | 25 (18, 33) | 0.427 | Yes |  | 1 | 0.17 |
| 2400-2800 | 31 (3367) | 5 | 32 (23, 41) | 0.092 | Yes |  | 1 | 2.45 |
| 2800-3200 | 7 (959) | 2 | 36 (25, 48) | 0.541 | Yes |  | 1 | 1.91 |
| 3200-3600 | 21 (1611) | 6 | 20 (13, 28) | 0.322 | Yes |  | 1 | 1.82 |
| 3600-4000 | 15 (459) | 4 | 42 (35, 49) | 0.028 | Yes |  | 1 | 2.23 |
| 4000-4400 | 11 (500) | 3 | 38 (29, 47) | 0.561 | Yes |  | 1 | 0.26 |
| 4400-4800 | 1 (500) | 0 | 19 (9, 29) | 0.537 | Yes |  | 1 | 0.27 |
| 4800-5200 | 7 (543) | 2 | 29 (7, 51) | 0.344 | Yes |  | 1 | 1.50 |
| 5200-5600 | 7 (1567) | 4 | 46 (24, 68) | 0.657 | Yes |  | 1 | 2.40 |
| 5600-6000 | 5 (1000) | 2 | 25 (4, 47) | 0.438 | Yes |  | 1 | 1.33 |
| 6000-6400 | 5 (3899) | 3 | 34 (20, 47) | 0.868 | Yes |  | 1 | 0.42 |
| 6400-6800 | 9 (2167) | 3 | 39 (31, 48) | 0.087 | Yes |  | 1 | 2.07 |
| 6800-7200 | 11 (1825) | 3 | 37 (28, 46) | 0.115 | Yes |  | 1 | 1.93 |
| 7200-7600 | 11 (2377) | 3 | 38 (31, 45) | 0.004 | Yes | Early stochastic | 1 | 0.19 |
| 7600-8000 | 19 (3497) | 4 | 33 (25, 41) | 0.041 | Yes |  | 1 | 1.71 |
| 8000-8400 | 11 (3409) | 3 | 38 (24, 52) | 0.497 | Yes |  | 1 | 0.36 |
| 8400-8800 | 9 (1851) | 5 | 30 (19, 42) | 0.173 | Yes |  | 1 | 2.71 |
| 8800-9200 | 7 (1889) | 3 | 32 (23, 41) | 0.1 | Yes |  | 1 | 1.67 |

Subject 686_Cl

| **Genomic region** | **No. haplotypes tested^a^** | **Max. HD**^b^ | **Poisson estimated tMRCA**^d^ | **GOF**^c^  **p-value** | **Star-like phylogeny** | **Explanation for deviation from model** | **No. founder viruses^f^** | **λ Poisson** |
| --- | --- | --- | --- | --- | --- | --- | --- | --- |
| E1-HVR1 (776) | 19 | 9 | 25 (14, 35)  22 (12,37) ^e^ | 0.103 | Yes |  | 1 | 2.152 |
| E2 (875) | 20 | 8 | 34 (27, 40)  11 (18,27) ^e^ | 0.742 | Yes |  | 1 | 4.105 |
| ***Analysis on reconstructed HCV variants across the genome in windows of 400 nucleotides*** | | | | | | | | |
| 1-400 | 59 (10971) | 6 | 34 (33, 35) | 0.073 | Yes |  | 1 | 1.23 |
| 400-800 | 23 (3031) | 2 | 35 (30, 39) | 0.001 | Yes | Early stochastic | 1 | 1.83 |
| 800-1200 | 43 (5591) | 4 | 34 (31, 38) | <10^-9^ | Yes | Early stochastic | 1 | 1.81 |
| 1200-1600 | 19 (3479) | 4 | 40 (35, 45) | <10^-9^ | Yes | Early stochastic | 1 | 2.10 |
| 1600-2000 | 43 (5039) | 4 | 37 (33, 41) | 0.657 | Yes |  | 1 | 0.58 |
| 2000-2400 | 27 (4873) | 4 | 32 (28, 36) | <10^-9^ | Yes | Early stochastic | 1 | 0.11 |
| 2400-2800 | 29 (4743) | 3 | 39 (36, 42) | 0.179 | Yes |  | 1 | 0.27 |
| 2800-3200 | 35 (5607) | 4 | 49 (35, 62) | 0.271 | Yes |  | 1 | 2.50 |
| 3200-3600 | 17 (1980) | 4 | 40 (34, 47) | 0.424 | Yes |  | 1 | 2.12 |
| 3600-4000 | 9 (989) | 4 | 38 (24, 52) | 0.497 | Yes |  | 1 | 0.37 |
| 4000-4400 | 9 (1005) | 4 | 41 (30, 53) | 0.505 | Yes |  | 1 | 2.18 |
| 4400-4800 | 9 (1653) | 4 | 42 (35, 50) | 0.108 | Yes |  | 1 | 2.23 |
| 4800-5200 | 9 (1295) | 4 | 34 (29, 40) | 0.002 | Yes | Early stochastic | 1 | 0.14 |
| 5200-5600 | 3 (950) | 2 | 25 (20, 31) | 0.438 | Yes |  | 1 | 0.14 |
| 5600-6000 | 13 (1561) | 2 | 30 (28, 32) | 0.005 | Yes |  | 1 | 0.16 |
| 6000-6400 | 11 (1600) | 3 | 38 (31, 46) | 0.365 | Yes |  | 1 | 0.24 |
| 6400-6800 | 7 (1361) | 2 | 32 (23, 41) | 0.1 | Yes |  | 1 | 1.67 |
| 6800-7200 | 9 (850) | 2 | 32 (23, 41) | 0.168 | Yes |  | 1 | 1.67 |
| 7200-7600 | 5 (989) | 2 | 17 (9, 26) | 0.2 | Yes |  | 1f | 0.05 |
| 7600-8000 | 7 (1335) | 4 | 34 (28, 40) | 0.659 | Yes |  | 1 | 0.25 |
| 8000-8400 | 11 (1503) | 3 | 38 (29, 47) | 0.039 | Yes |  | 1 | 2 |
| 8400-8800 | 7 (1357) | 3 | 43 (28, 59) | 0.564 | Yes |  | 1 | 0.823 |

Subject 360_Cl

| **Genomic region** | **No. haplotypes tested^a^** | **Max. HD**^b^ | **Poisson estimated tMRCA**^d^ | **GOF**^c^  **p-value** | **Star-like phylogeny** | **Explanation for deviation from model** | **No. founder viruses^f^** | **λ Poisson** |
| --- | --- | --- | --- | --- | --- | --- | --- | --- |
| E1-HVR1  (680) | 18 | 7 | 20 (11, 30)  8 (4,14) ^e^ | 0.015 | Yes |  | 1 | 0.4292 |
| E2 (932) | 18 | 5 | 14 (9, 19)  8 (4,13) ^e^ | 0.840 | Yes |  | 1 | 0.297 |
| ***Analysis on reconstructed HCV variants across the genome in windows of 400 nucleotides*** | | | | | | | | |
| 1-400 | 29 (3621) | 7 | 31 (23, 38) | 0.438 | Yes |  | 1 | 1.33 |
| 400-800 | 9 (779) | 4 | 27 (13, 41) | 0.344 | Yes |  | 1 | 3.27 |
| 800-1200 | 35 (2275) | 7 | 30 (24, 35) | 0.339 | Yes |  | 1 | 2.94 |
| 1200-1600 | 31 (3113) | 7 | 44 (29, 58) | 0.613 | Yes |  | 1 | 2.238 |
| 1600-2000 | 17 (1214) | 5 | 48 (39, 56) | 0.250 | Yes |  | 1 | 2.64 |
| 2000-2400 | 15 (1727) | 6 | 53 (44, 61) | 0.710 | Yes |  | 1 | 2.74 |
| 2400-2800 | 15 (2353) | 5 | 36 (28, 44) | 0.170 | Yes |  | 1 | 1.974 |
| 2800-3200 | 21 (2303) | 6 | 49 (40, 57) | 0.577 | Yes |  | 1 | 2.686 |
| 3200-3600 | 53 (7927) | 6 | 25 (21, 29) | 0.003 | Yes | Early stochastic | 1 | 1.386 |
| 3600-4000 | 27 (4619) | 6 | 25 (20, 30) | 0.124 | Yes |  | 1 | 1.376 |
| 4000-4400 | 27 (2641) | 4 | 20 (15, 26) | 0.474 | Yes |  | 1 | 1.1 |
| 4400-4800 | 15 (3033) | 4 | 49 (39, 58) | 0.160 | Yes |  | 1 | 2.7 |
| 4800-5200 | 33 (4589) | 8 | 33 (27, 40) | 0.0002 | Yes | Early stochastic | 1 | 1.846 |
| 5200-5600 | 11 (837) | 3 | 36 (27, 46) | 0.113 | Yes |  | 1 | 2 |
| 5600-6000 | 7 (1541) | 5 | 50 (37, 63) | 0.56 | Yes |  | 1 | 2.76 |

^a^ Variants in windows of 400 nucleotides.

^b^ HD: Hamming distance

^c^ GOF: Goodness of fit

^d^ Shown are the mean value and the confidence interval obtained from the PoissonFitter program.

^e^ Estimates from BEAST using coalescent model. See details in the text.

^f^ Number of viruses estimated from phylogenetic analysis.
