# Supplementary material for: Sequential Bottlenecks Drive Viral Evolution in Early Acute Hepatitis C Virus Infection
Source: PLoS Pathog. 2011 Sep 1;7(9):e1002243. doi: 10.1371/journal.ppat.1002243 (PMC3164670; doi:10.1371/journal.ppat.1002243)
Supplement: Table S5 — Analysis of amino acid substitutions within predicted HLA restricted cytotoxic T cell epitopes. (DOC) [file ppat.1002243.s010.doc]

**Table S5. Analysis of amino acid substitutions within predicted HLA restricted cytotoxic T cell epitopes.**

| Subject | Mutations in epitopes^a^ | Mutations reducing HLA affinity^b^ | Total number of fixations | Fixations occurring in epitopes |
| --- | --- | --- | --- | --- |
| 23_Ch | 29 | 10 (34%) | 4 | 4 |
| 240_Ch | 12 | 4 (33%) | 18 | 9 |
| 686_Cl | 8 | 0 (0%) | 0 | - |
| 360_Cl | 13 | 2 (15%) | 0 | - |

^a^ Only substitutions with a frequency of occurrence above 1% are included.
