# Supplementary material for: Sequential Bottlenecks Drive Viral Evolution in Early Acute Hepatitis C Virus Infection
Source: PLoS Pathog. 2011 Sep 1;7(9):e1002243. doi: 10.1371/journal.ppat.1002243 (PMC3164670; doi:10.1371/journal.ppat.1002243)
Supplement: Table S6 — Primers used to amplify viral sequences. (DOC) [file ppat.1002243.s011.doc]

**Table S6. Primers used to amplify viral sequences.**

| **Region** | **Round** | **Direction** | **Genotype** | **Primer name** | **Sequence (5'-3')** |
| --- | --- | --- | --- | --- | --- |
| 5'UTR-NS3 | Outer | + | All | hep14 | GCAGAAAGCGTCTAGCCATGGCGT |
|  |  | - | 1a | GV187 | TCGGACCTTTACYTGGTCACGAG |
|  |  | - | 2b/3a | hep343 | GGRATGACRTCRGCATCGCGGGTAACCA |
|  | Inner | + | All | hep21b | GAGTGTYGTRCAGCCTCCAGG |
|  |  | - | All | hep344 | GTYTGCTGRGCGTAYGCMGTGATGGG |
| NS2-NS5B | Outer | + | 1a | hep299 | ATGGAGACCAAGCTCATCACGTGGG |
|  |  | + | 2b | hep319 | ATGGAGAAGAARGTCATYGTSTGGG |
|  |  | - | 1a | hep109 | ACGGAATTCGCGGGGTCGGGCVYGNGACA |
|  |  | - | 2b | hep323 | GGAGTGTASCTARTGTGTGCCGCT |
|  | Inner | + | 1a | hep300 | GGGGGCAGATACCGCCGCGTGCGG |
|  |  | + | 2b | hep320 | GGGGGCKGAGACRGCRGCWTGTGG |
|  |  | - | All | hep296 | CGGGCAYGAGACASGCTGTGATAWATGTC |
| Core-p7 | Inner | + | All | GV34 | TGCGGMTTCGCCGACCTCATGG |
|  |  | - | 1a | hep310 | CCYTGRACGCGCACRAAGTAGGG |
|  |  | - | 2b | Hep314a | CAGCCACCACACYGAACGGC |
|  |  | - | 3a | hep318 | GCGCTGTCTTCACCCGACCA |
| NS2-4B | Outer | + | 1a | hep299 | ATGGAGACCAAGCTCATCACGTGGG |
|  |  | + | 3a | hep301 | ATGGAAATCAAGGTCATCACCTGGG |
|  |  | - | 1a | GV192 | GCTATYAGCCGGTTCATCCAYTGCAC |
|  |  | - | 3a | hep303 | GCGATGAGCCTGTTCATCCA |
|  | Inner | + | 1a | hep300 | GGGGGCAGATACCGCCGCGTGCGG |
|  |  | + | 3a | hep302 | TGGGGCGCGGATACRGCRGCTTGCGG |
|  |  | - | 1a | GV193 | GCRTCGCTCTCNGGCACRTAGTG |
|  |  | - | 3a | hep304 | GCAGCATCGCTCTCGGGAACATAGTG |
| NS4B-5B | Outer | + | 1a | hep255 | GTGCARTGGATGAACCGGC |
|  |  | + | 3a | hep351 | GTCCAGTGGATGAACAGGC |
|  |  | - | 1a | hep109 | ACGGAATTCGCGGGGTCGGGCVYGNGACA |
|  |  | - | 3a | hep234b | TGGAGTGTTATCYTACCAGC |
|  | Inner | + | 1a | hep256 | TCCCCCACRCACTAYGTGCC |
|  |  | + | 3a | hep352 | TCACCRACGCACTATGTTCCCG |
|  |  | - | All | hep296 | CGGGCAYGAGACASGCTGTGATAWATGTC |
