# Supplementary material for: Sequential Bottlenecks Drive Viral Evolution in Early Acute Hepatitis C Virus Infection
Source: PLoS Pathog. 2011 Sep 1;7(9):e1002243. doi: 10.1371/journal.ppat.1002243 (PMC3164670; doi:10.1371/journal.ppat.1002243)
Supplement: Text S1 — Haplotype reconstruction and method validation. Detailed information on the haplotype reconstruction from NGS data. This methodology was validated with a 454 FLX run performed in duplicate on four unique plasmids with known sites of variation. (DOC) [file ppat.1002243.s012.doc]

**Supporting Text 1: haplotype reconstruction and method validation**

This section contains detailed information on the analysis for haplotype reconstruction from NGS data. This methodology was validated with a 454 FLX run performed in duplicate on four unique plasmids with known sites of variation.

**Study design**

Four plasmids carrying the HCV Envelope region inserted into pGEM-T Easy vector obtained from a single viremic time point from subject 23_Ch were purified and mixed in estimated equal molar proportions (25%) in a single tube. The DNA was quantified with the Quant-iT PicoGreen dsDNA Kits (Invitrogen). This was submitted to 454 Roche NGS. Each variant in the mixture sample included a 2572 nt region of the HCV genome (Core-E-p7), and 2600 nt of the vector, for a total of 5172 nt. Results of SNP analyses are in Table S2.

The goal of this experiment was to test whether the method based on Bayesian analysis of NGS data (ShoRAH), successfully reconstructed the four known variants and accurately calculated the frequency of each plasmid within the sample (25%). For comparison another commonly utilized method for SNP detection based on a cut-off value was employed, VARSCAN [3].

**Validation of SNP detection**

Automated cleaning (as explained in the Materials and Methods) eliminated most of the spurious SNPs (~70-80% of the false positives). The remaining false positive SNPs were at a frequency lower than 1% within the sample and mostly within homopolymeric stretches. Further manual checking of these regions was required to eliminate those errors. The error rate in the E1-E2 region (1700 nt) after automated cleaning, and before manual cleaning, with a detection threshold of 1% was 0.0075; while for a detection threshold of 0.1%, the error rate was 0.01875. The error rate in the vector region before automated cleaning with a detection threshold of 0.1% was 0.0123, whereas with a detection threshold of 1% the error rate was 0.00769. These estimates are consistent with previous reports using Roche 454 data to analyse low frequency viral variants [4].

A conservative procedure involving a manual check of aligned reads was employed, as proposed elsewhere [4,5]. Variants present within homopolymeric regions with frequencies <1% and present mainly in the forward (or mainly in the reverse) reads, were replaced with the consensus nucleotide. Also, reads with hypermutated ends (>5 nucleotides mutated in the last 20 nt) were also removed. With this manual procedure only true SNPs in the plasmids were detected across the E1-E2 region and no variants detected in the vector region, as expected (See Table).

**Table.** SNP detection in the control NGS run performed on a mixture of four HCV plasmids prepared from subject 23_Ch at time point (44 DPI).

| **Detection method** | **Total number of SNPs (>0.1%)** | **Total number of SNPs (>1%)** | **Number of true SNPs** |
| --- | --- | --- | --- |
| ***HCV plasmid E1-E2 region*** | | | |
| Varscan (cut-off) | 52 | 48 | 34 |
| ShoRAH (Bayesian) | 64 | 46 | 34 |
| ShoRAH (manually curated) | 34 | 34 | 34 |
| ***Vector pGEM-T Easy region*** | | | |
| Varscan (cut-off) | 29 | 13 | 0 |
| ShoRAH (Bayesian) | 32 | 20 | 0 |
| ShoRAH (manually curated) | 0 | 0 | 0 |

**Validation of haplotype reconstruction.** Along with SNP detection, the Bayesian method implemented within ShoRAH also allows reconstructing haplotypes with sequence lengths greater than the average length of a 454 read. Here ShoRAH was used to reconstruct the Envelope region of the genome (~1600 nt).

The figure below shows a comparison between the four sequences of the Envelope region used in the mixture sample and the four reconstructed variants obtained from ShoRAH using a highlighter plot (Los Alamos National Laboratory, <http://www.lanl.gov/content/sequence/HIGHLIGHT/highlighter.html>). This analysis was performed in triplicate with only sequences reconstructed in all three runs shown in Fig. S1. The same analysis also revealed a perfect match of the reconstructed vector with the true vector sequence. For this analysis, only reconstructed variants with frequencies >2.5% within the sample were used. Below this frequency threshold, chimeras were observed. As shown in the highlighter plot (see figure below), the four variants were estimated at frequencies 34%, 22%, 11%, and 24%, respectively. This result was confirmed on a second run of 454 FLX using the same library preparation (data not shown). Three mutated sites - with expected mutations of 25% - were found with frequency of mutants between 10-15%. The lowest estimate of 11% was due to either a technical error during sample preparation or during the 454 library preparation, which likely caused a disproportionate molar ratio of individual plasmids in the run. This estimate was considered unlikely to be due to a ShoRAH error, but rather to a technical error in sample preparation. The standard cut-off method also showed similar underestimated frequencies.

For the application of this methodology in the analysis of the four early infection subjects, smaller sections (E1-HVR1, and E2) within the Envelope region were reconstructed. This restriction was used as a conservative approach, because although we successfully reconstructed ~1600 nt of clonal sequence, these variants were present at high frequency. In reality, rare variants are likely to exist *in vivo*, and some of those are likely to carry low diversity, which can limit the accuracy of reconstruction via ShoRAH. The E1/HVR1 and E2 regions contain the most variable regions within the genome, hence maximizing the accuracy of reconstructions. More details of the clustering algorithm employed in ShoRAH are given in Zagordi *et al.* [1,2].

**
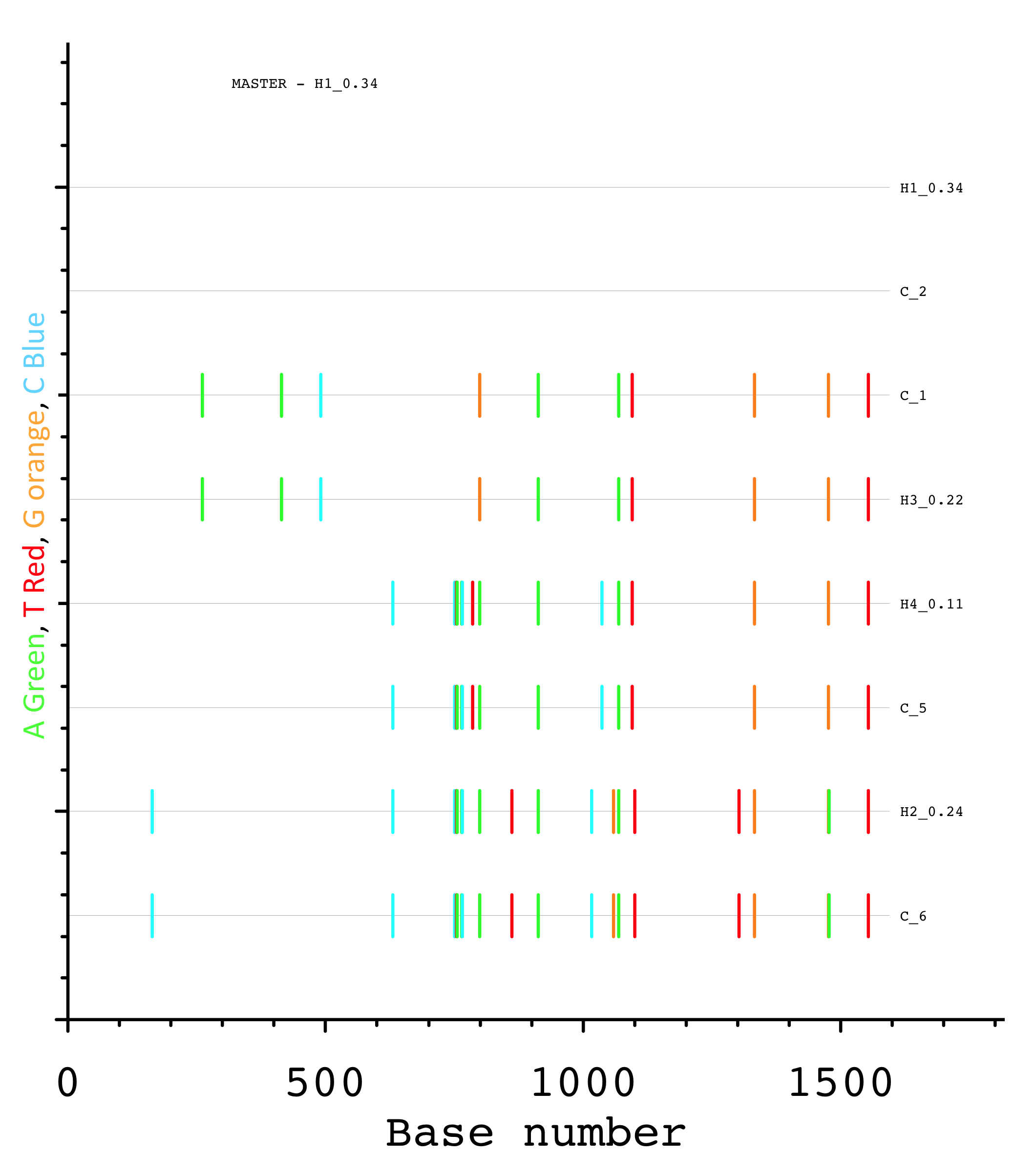
**

**Figure.** Highlighter plot (Los Alamos National Laboratory website - HIV Sequence Database) showing the identity between sequences from four distinct plasmids used in the mixed variant experiment (C1, C2, C3, C4) and the four reconstructed haplotypes derived from 454 reads using ShoRAH. Names of reconstructed haplotypes carry the estimated frequencies of the 4 plasmids in the mixed sample.

# References

1. Zagordi O, Klein R, Daumer M, Beerenwinkel N (2010) Error correction of next-generation sequencing data and reliable estimation of HIV quasispecies. Nucleic Acids Res 38: 7400-7409.

2. Zagordi O, Geyrhofer L, Roth V, Beerenwinkel N (2010) Deep sequencing of a genetically heterogeneous sample: local haplotype reconstruction and read error correction. J Comput Biol 17: 417-428.

3. Koboldt DC, Chen K, Wylie T, Larson DE, McLellan MD, et al. (2009) VarScan: variant detection in massively parallel sequencing of individual and pooled samples. Bioinformatics 25: 2283-2285.

4. Tsibris AM, Korber B, Arnaout R, Russ C, Lo CC, et al. (2009) Quantitative deep sequencing reveals dynamic HIV-1 escape and large population shifts during CCR5 antagonist therapy in vivo. PLoS One 4: e5683.

5. Fischer W, Ganusov VV, Giorgi EE, Hraber PT, Keele BF, et al. (2010) Transmission of single HIV-1 genomes and dynamics of early immune escape revealed by ultra-deep sequencing. PLoS One 5: e12303.
